# A bibliometric method for assessing technological maturity: the case of additive manufacturing

**DOI:** 10.1007/s11192-018-2941-1

**Published:** 2018-11-01

**Authors:** René Lezama-Nicolás, Marisela Rodríguez-Salvador, Rosa Río-Belver, Iñaki Bildosola

**Affiliations:** 1 Escuela de Ingeniería y Ciencias, Tecnologico de Monterrey, Ave. Eugenio Garza Sada 2501, 64849 Monterrey, N.L. Mexico; 2 Foresight, Technology and Management (FTM) Group, Industrial Organization and Management Engineering Department, University College of Engineering of Vitoria-Gasteiz, University of the Basque Country UPV/EHU, Basque Country, Leioa, Spain; 3 Foresight, Technology and Management (FTM) Group, Industrial Organization and Management Engineering Department, Faculty of Engineering in Bilbao (ETSI Bilbao), University of the Basque Country UPV/EHU, Basque Country, Leioa, Spain

**Keywords:** Technology maturity, Technology readiness level, Technology life cycle, Bibliometrics, Additive manufacturing, 62-07, C13

## Abstract

While novel technologies have tremendous competitive potential, they also involve certain risks. Maturity assessment analyzes how well a technological development can fulfill an expected task. The *technology readiness level* (TRL) has been considered to be one of the most promising approaches for addressing technological maturity. Nonetheless, its assessment requires opinions of the experts, which is costly and implies the risk of personal bias. To fill this gap, this paper presents a *Bibliometric Method for Assessing Technological Maturity* (BIMATEM). It is a repeatable framework that assesses maturity quantitatively. Our method is based on the assumption that each technology life cycle stage can be matched to technology records contained in scientific literature, patents, and news databases. The scientific papers and patent records of mature technologies display a logistic growth behavior, while news records follow a hype-type behavior. BIMATEM determines the maturity level by curve fitting technology records to these behaviors. To test our approach, BIMATEM was applied to additive manufacturing (AM) technologies. Our results revealed that *material extrusion, material jetting, powder bed fusion* and *vat photopolymerization* are the most mature AM technologies with TRL between 6 and 7, followed by *directed energy deposition* with TRL between 4 and 5, and *binder jetting* and *sheet lamination*, the least mature, with TRL between 1 and 2. BIMATEM can be used by competitive technology intelligence professionals, policymakers, and further decision makers whose main interests include assessing the risk of implementing new technologies. Future research can focus on testing the method with regard to altmetrics.

**Electronic supplementary material:**

The online version of this article (10.1007/s11192-018-2941-1) contains supplementary material, which is available to authorized users.

## Introduction

Under the dynamics of the current market environment, technological innovations represent more isolated competitive advantages. They have become a *necessity* that drives quality to the limits of perfection as the marketplace is filled with more competitors and product life cycles are shortened. Technological innovations may provide an organization with several benefits. Acquiring or developing the right innovation might expand infrastructural capabilities, increase strategic options, boost efficiency, and help a firm to respond more promptly to the competitive environment (Mortara and Ford 2012).

**Fig. 1:**
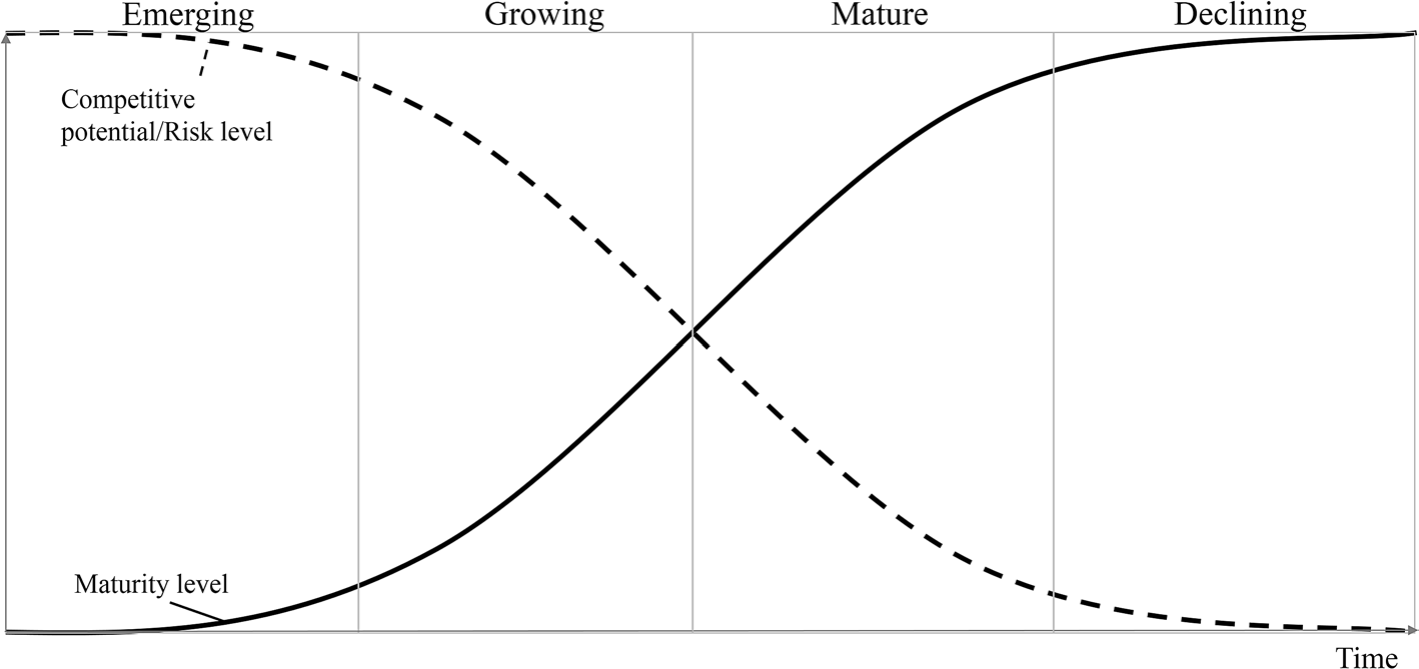
Technology life cycle (TLC) stages. Adapted from (Ansoff and McDonnell 1984; Ernst 1997; Reinhart and Schindler 2010)

However, the implementation of new technologies involves complex challenges as myriads of technological solutions are available in the market. These solutions must be carefully assessed for strategic and operational planning processes. Placing immature technologies into products can generate risks associated with cost, schedule, and performance, while implementing them in manufacturing processes can result in low yield, high defect rates, rework, and hand work during production (Nolte 2008). Conversely, technologies that are considerably mature (in decline) may be counterproductive. As the market becomes saturated with mature technologies, the competitive potential decreases (Reinhart and Schindler 2010). The technology life cycle (TLC) shown in Fig. 1 exhibits this behavior in which a continuous ascending line represents the pace at which the maturity increases. Inversely, a descending dotted line depicts how the competitive potential and risk level decrease. This model reflects the negative correlation between maturity and competitive potential/risk levels.

It is crucial to identify the best technological option. For this purpose, it is important to have a *risk* indicator for assessing the new technology to be implemented. For years, maturity assessment has been regarded as a risk indicator that serves this purpose (Engel et al. 2012). The TLC has been proven to be an essential model for comprehending the state of technological maturity (Ardilio et al. 2012). Herein, technology is conceptualized as a cycle wherein capabilities and competitiveness arise and decay over time. In an analogy to the biological life cycle, this cycle reveals how technology progresses through various stages, including birth (emerging), childhood (growing), adulthood (maturity), elderly (decline), and death, when new technologies replace the previous ones to repeat the cycle again. The speed at which technology traverses through the life cycle depends on the capability to overcome technical challenges (Roper et al. 2011). In this sense, a mobile phone technology may reach maturity within three years, while an automotive technology may require up to 15 years to reach maturity (Ardilio et al. 2012).

Maturity is defined as the stage in the TLC wherein a technology has been sufficiently developed to meet its required performance (Choi et al. 2013). A mature technology is a technology that is well understood and fully controlled, such as bicycle gearing or vapor compression that is used in most cooling systems. The more mature a technology is, the safer it is to implement it into product development. Conversely, an immature technology is the technology that has not been sufficiently developed and that may behave unexpectedly, such as the internal configuration of the lithium-ion batteries that caused *Samsung Galaxy Note 7™* phones to explode (Lloyd 2017) and generated an approximated loss of $5.3 billion in recall costs (Baig 2016).

Estimating technological maturity is typically approached using expert-based methods (Albert 2016), such as Delphi or brainstorming (Lee et al. 2017). This notion of formation is usually considered as a shortcoming for its lack of repeatability, reliability and objectivity (Albert 2016) since there is an inherent risk of personal bias in the assessment. Additionally, this approach cannot guarantee efficiency because contacting or gathering experts may be costly and time-consuming.

There have been techniques that gauge technological maturity without the assessment of experts, primarily by measuring a technological parameter and assessing its change over time. Kayal (1999) selected the median age of patent cited in patent applications, asserting that the shorter the time, the more mature the technology. This approach was later considered naïve (Martino 2003) since single parameters (such as the median) tend to be insufficient for most technologies.

Additionally, there have been proposals to analyze technological maturity through patent indicators (Haupt et al. 2007). For instance, Gao et al (2013) proposed a method based on multiple patent indicators to assess technological progress in the TLC. However, such proposals involved expert assessment to some extent, since researchers were required to choose technologies similar to the ones being tested.

To address the aforementioned shortcomings of assessing technological maturity, the US National Aeronautics and Space Administration (NASA) created the *technology readiness level* (TRL) scale in the 1970 s (Mankins 2009). This is a well-defined scale that assesses technological maturity by proving technical capabilities (US Government Accountability Office 2016).

The TRL has been the most accepted approach to determine technological maturity (Olechowski et al. 2015). It received global recognition in the 1990s when the official nine-level TRL was published (Mankins 1995) as an unprecedented tool for assessing technological maturity on a standardized numerical scale. US federal organizations and numerous private companies have adopted it as a regular planning and assessment tool (European Association of Research and Technology Organisations 2014). The TRL has been noted for its ability to systematically communicate the readiness of new applications to be incorporated into a product and provide a common language for technology developers, program managers, and acquisition officials (US Government Accountability Office 2016). Table 1 summarizes the original TRL definition and its adaptations to different organizations’ perspectives.

**Table 1:** The definitions and classifications of the technology readiness level (TRL)

TRL	Original definitions of TRLs (Mankins 1995)	European Commission’s TRL definition (European Commission 2014)	Maturity cluster (European Association of Research and Technology Organisations 2014)	System fidelity (Sanchez 2015)
1	Basic principles observed and reported	Basic principles observed	Invention	System exists on paper (no hardware system)
2	Technology concept and/or application formulated	Technology concept formulated		
3	Analytical and experimental critical function and/or characteristic proof of concept	Experimental proof of concept	Concept validation	System matches a piece or pieces of the final application
4	Component and/or breadboard validation in laboratory environment	Technology validated in laboratory		
5	Component and/or breadboard validation in relevant environment	Technology validated in relevant environment (industrially relevant environment in the case of key enabling technologies)	Prototyping and incubation	System matches final application in almost all respects
6	System/subsystem model or prototype demonstration in relevant environment (ground or space)	Technology demonstrated in relevant environment (industrially relevant environment in the case of key enabling technologies)	Pilot production and demonstration	
7	System prototype demonstration in a space environment	System prototype demonstration in operational environment		
8	Actual system completed and “flight qualified” through test and demonstration (ground or space)	System complete and qualified	Initial market introduction	System matches final applications in all respects
9	Actual system “flight proven” through successful mission operations	Actual system proven in operational environment (competitive manufacturing in the case of key enabling technologies; or in space)	Market expansion	

Despite its recognition for placing technological maturity on a numerical scale, the TRL might be influenced by subjective perspectives. This is because the TRL is mostly assessed through expert surveys (Engel et al. 2012). To fill this gap, different qualitative and quantitative proposals have been developed. Nolte et al. (2003) created a TRL calculator that is based on a *Microsoft Excel* spreadsheet and programmed using *Visual Basic* macros. Terrile et al. (2015) proposed another solution by calibrating the TRL with the cost data of NASA’s project milestones. They plotted cumulative project costs and revealed an S-shaped curve wherein TRLs were accordingly matched as the curve progressed. This proposal establishes a more objective metric: monetary units. However, their results were adjusted to schedule variation within NASA’s framework. To transform this approach into a viable solution, additional project data and tests by other organizations are required to be incorporated into their proposal to define acceptable programmatic variance. Additionally, Wei-gang et al. (2013) proposed a solution in which TRLs were correlated with quantitative technological parameters, such as working hours, failure frequency, or repair time. This method is appealing since it depicts each TRL based on operational variables. However, this approach does not eliminate the risk of personal bias since it requires experts to define the aforementioned parameters.

Although TRL is highly valuable for its quantitative output, it is qualitatively determined using non-repeatable methods, primarily via expert opinions. According to Albert (2016), these shortcomings may be addressed through standardization of methods (where a uniform approach is consistently deployed to assure repeatability), operationalization (where measurable information is obtained), and automation (where efficiency is injected to the process and most human interaction is minimized along the process).

This research aims to address these shortcomings by providing a standardized technique that quantitatively estimates the level of technological maturity in a semi-automated manner. Our approach is based on bibliometric analysis of records of mature technologies. We constructed a methodology based on Watts and Porter’s (1997) approach of bibliometric estimators concerning the stages of research and development (R&D) progress. Our method incorporates Wong and Goh’s (2010) findings on the logistic growth behavior of science and technology records (scientific papers and patents) of mature technologies. We enriched it with a finding regarding the hype-type behavior (Campani and Vaglio 2015) in news records of mature technologies.

This section shed light on the importance of analyzing maturity as a measure of assessing the risk associated with the implementation of new technologies. Additionally, it introduced the concept of TLC, a model for understanding technology progression, and the benefits and setbacks of TRL, one of the most extended approaches for assessing technological maturity.

The remainder of this study is organized as follows. “Background” section merges the concepts of the TLC and TRL with bibliometrics to track the innovation progress: from basic research to applied research to product development (Godin 2006). It also covers the mathematical background concerning the logistic growth behavior for science and technology records as well as the hype-type behavior for news records. “BIMATEM” section describes the *Bibliometric Method for Assessing Technological Maturity* (BIMATEM), the technique proposed to assess technological maturity, wherein records of a given technology are output to the TRL. “Case of application: AM technologies” section applies BIMATEM to the seven additive manufacturing (AM) technologies that are officially recognized by the American Society for Testing and Materials (ASTM) and assigns a maturity level to each of them. “Results and discussion” section discusses the findings and explores the implications that BIMATEM could have in different organizations. Conclusion section summarizes the method, presents its limitations, and states future work.

## Background

**Fig. 2:**
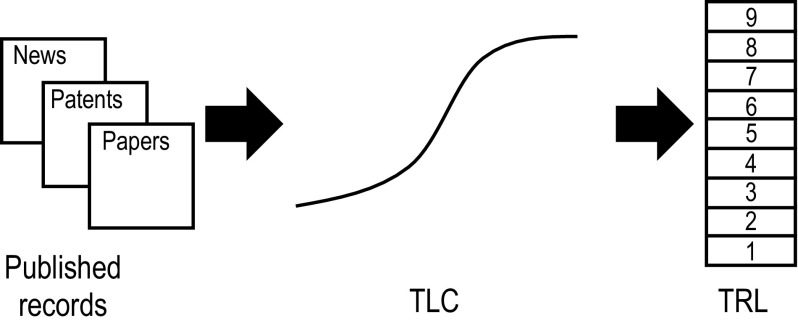
Proposed approach to obtain the technology readiness level (TRL)

This paper aims to present a repeatable, reliable and semi-automated method for estimating technological maturity via TRL. To this end, we proposed to estimate the TRL as an approximation of TLC stages, which in turn can be obtained through bibliometrics records, as shown in Fig. 2.

Bibliometrics corresponds to the statistical analysis of publications (OECD 2013). It has been used by researchers, governments and organizations to explore large amounts of publications to identify patterns that aid decision making. Examples of its application range from exploratory analyses on research sectors (Bornmann and Leydesdorff 2014), technology forecasting (Daim et al. 2006), and more recently, sentiment analysis at both industrial and corporate levels (Garechana et al. 2017).

One of the most exploited bibliometric indicators is the number of publications (Okubo 1997). This indicator is typically performed through text mining techniques, also known as data mining or “tech mining” when applied to science and technology documents (Porter and Cunningham 2005). Herein, large volumes of data are filtered and processed to determine specific bibliometric indicators.

With regards to the assessment of technological maturity, bibliometrics has been used as an operationalized approach to estimate it (Albert 2016). A method for assessing technological maturity through bibliometrics, has been created by approximating the number of publications to different stages along the linear model of innovation. This model postulates that technology starts with basic research, which is “performed without thought of practical ends (…) and results in general knowledge and an understanding of nature and its laws” (Bush 1945). This stage then evolves into applied research, namely the research focused on solving practical problems (Palys 2008). Afterwards, it turns into development, where new products and processes can be industrially created from it (Godin 2006). Finally, it reaches the diffusion stage where technology reaches the market (Schumpeter 1939).

The linear model of innovation has been criticized for its linear nature (Kline 1985). However, it is a model that has remained valid (despite criticism) for over fifty years because it has permitted an easy tracking of innovation evolution (Godin 2006).

Watts and Porter (1997) proposed a method to estimate technological maturity based on the linear model of innovation. They selected specific scientific, technology, and news databases to match the TLC stages, as summarized in Table 2, and indicated that a certain amount of records would rise and peak in every bibliometric source as each TLC stage was attained. We updated these bibliometric sources considering those that we found more suitable for tech mining. In addition, we also matched each TLC stage to a TRL.

**Table 2:** TRL from technology life cycle (TLC) stages obtained through publications Adaptedfrom Watts and Porter (1997)

TLC stages	Bibliometric sources	Databases	TRL
Emerging	N/A	N/A	1
			2
	Scientific papers	Science Citation Index™ (Clarivate Analytics 2017a)	3
	Engineering papers	EiCompendex™ (Elsevier 2017)/INSPEC™ (Clarivate Analytics 2017a)/MEDLINE™ ^a^ (Clarivate Analytics 2017a)	4
			5
Growing	Patents	PATENTSCOPE™ (WIPO 2017c)/USPTO (USPTO 2017)/Espacenet (EPO 2017)/Patseer™ (Gridlogics Technologies 2017)	6
			7
Mature	News records	Factiva™ (Dow Jones 2017)	8
			9

^a^For life science technologies

The *emerging* TLC stage corresponds to TRLs 1–5 when technology concepts are observed (TRL 1), formulated (TRL 2), experimented (TRL 3), validated in the laboratory (TRL 4), and validated in a relevant environment (TRL 5). TRLs 1 and 2 are not linked to any bibliometric source because these steps belong to a nascent stage along the scientific method. Most scientific journals require proof of concepts through experimentation for publication. Records pertaining to TRL 3 may be found in the *Science Citation Index™* that covers multidisciplinary scientific articles since 1900 (Clarivate Analytics 2017a). Documents corresponding to applied (engineering) research (TRLs 4 and 5) can be found in data collections, such as *EiCompendex™* (an engineering-specific literature database), which indexes records since 1970 (Elsevier 2017), *INSPEC™* (produced by the Institution of Electrical Engineers), which focuses on engineering and technical research (Clarivate Analytics 2017a) and contains records since 1969, or *MEDLINE™* (from the U.S. National Library of Medicine), which indexes records concerning life sciences since 1950 (Clarivate Analytics 2017a).

The *growing* TLC stage corresponds to TRLs 6 and 7 when prototypes are demonstrated. Herein, patent databases are suited to prove the achievement of these TRL stages. Some of the patent databases that we recommend are *PATENTSCOPE* [which belongs to the World Intellectual Property Organization (WIPO 2017c)], the *United States Patent and Trademark Office* database (USPTO 2017), *Espacenet* (the European Patent Office patent data collection), or *Patseer™* [a commercial database that covers these previous patent databases among over 61 million full text records of 43 authorities; (Gridlogics Technologies 2017)]. These patent databases index records prior to the 20th century.

Finally, the *mature* TLC stage is linked to TRLs 8 and 9 when technology is proven, qualified, and implemented in an operational environment. News databases—such as *Factiva™* (Dow Jones 2017), which covers over 32,000 sources from 1951 to the present, including newspapers, journals, magazines, and blogs—are used to retrieve records corresponding to this TLC stage. The *decline* TLC stage does not appear in Table 2 because this phase—where competitive potential is lost—goes beyond the TRL-intended assessment.

As each bibliometric database has been defined as a proxy to estimate the TRL, the next step is to define an estimation parameter to test them. Originally, the rising and declining (peak) of technology publications was used as the estimator parameter. Nevertheless, this was eventually proven fallacious as the publications of science and technology tend to self-propagate (Watanabe et al. 2003). Although most research recognizes that the diffusion of technological innovation evolves by following an approximate logistic growth behavior (S-shaped curve) (Nieto et al. 1998), empirical testing (Järvenpää et al. 2011) suggests that this is true when science is the technological driver (i.e. technology push, as opposed to market pull).

The logistic growth behavior begins gradually and progressively accelerates to pass an inflection point (modeled in the middle in Fig. 1), where it starts to decelerate until it finally reaches stagnation. Its behavior can be mathematically represented as follows:

1 $$r\left( t \right) = \frac{k}{{1 + ae^{{ - b\left( {t - t_{0} } \right)}} }}$$

where *k* is the upper limit to the growth of *r(t)*, also known as the carrying capacity. The initial stage of diffusion is represented by *a*, and *b* is the velocity of diffusion.

However, news records do not display a trend corresponding to the logistic growth function. A bibliometric analysis of news records is severely lacking in the literature. To fill this gap, we tested publication frequencies from 10 mature technologies in *Factiva™*, the news database. These technologies were identified by Fenn (2014) and are listed in Table 3. To retrieve the most accurate results, synonyms were included in the search strategy. The search query was launched in the headline field. Further details concerning the query in *Factiva™* are provided in Table A1.3 and A1.3.1 in Appendix of Electronic Supplementary Material 1.

**Table 3:** A total of 10 mature technologies Adapted from (Fenn 2014) and defined by Gartner Inc. (2017)

Technology	Synonyms and syntax variations	Definition (Gartner Inc. 2017).
Cloud computing	N/A	“Style of computing in which scalable and elastic IT-enabled capabilities are delivered as a service using Internet”
Datamining	Data-mining, data mining, text-mining, textmining, and text mining	“The process of discovering meaningful correlations, patterns and trends by sifting through large amounts of data stored in repositories”
Location-aware technology	Location intelligence technology	“Sensors and methods for detecting or calculating the geographical position of a person, a mobile device or other moving objects”
Microelectromechanical systems	MEMS and microelectronic systems	“Semiconductor devices incorporating structures that can physically move, in addition to electronic circuits”
Organic light-emitting diode	OLED and organic light-emitting device	“LED with an emissive electroluminescent layer made from organic compounds”
Radio-frequency identification	RFID	“Devices that respond to a reader’s interrogation via radio frequency”
Smartphone	Smart-phone and smart phone	“Mobile communications device that uses an identifiable open operating system”
Speech recognition	Speech to text	Systems that “interpret human speech and translate it into text or commands”
Text to speech	Speech synthesis and text to voice	“Technology that converts text into spoken audiostream”
Wireless local area network	Wireless LAN, WLAN, Wi-Fi, IEEE 802.11, IEEE STD 802.11, and IEEE 802 standard	“LAN communication technology in which radio, microwave or infrared links take the place of physical cables”

**Fig. 3:**
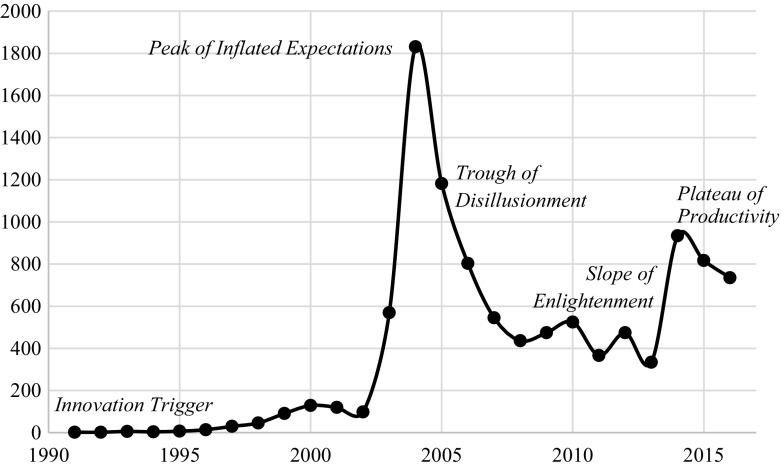
*Factiva™* records of radio-frequency identification (*RFID*). They display the characteristic hype-type behavior (Fenn et al. 2013) given by the *innovation trigger, peak of inflated expectations, trough of disillusionment, slope of enlightenment*, and *plateau of productivity*

News records of mature technologies exhibit a hype-type behavior. This behavior has been depicted in Gartner’s *Hype Cycles* (Fenn et al. 2013) since 1995. It is formed by merging a market expectations equation in the form of a Gaussian bell, and a logistic growth curve revealing technological maturity (Dedehayir and Steinert 2016; Steinert and Leifer 2010). According to this model, technologies start from an *innovation trigger*, where the initial media interest starts but no real products have been developed from it. Then, it reaches a *peak of inflated expectations* characterized by a hype of success stories. The next factor that is considered is the *trough of disillusionment* as the previously hyped applications fail to comply when technology is implemented in general industries: the technology is yet to overcome certain challenges. Next, a *slope of enlightenment* appears as the realistic applications and best practices of the technology use are attained. Finally, the *plateau of productivity* represents the initiation of the mainstream adoption. We exemplify the hype-type behavior through publication records of radio-frequency identification (*RFID*) in *Factiva™* (Fig. 3).

The hype-type behavior was remarkably modeled by Campani and Vaglio (2015) as the superposition of the functions Q(t) and S(t′).

2 $$H\left( t \right) = Q\left( t \right) + S\left( {t^\prime } \right)$$

Q(t) reveals the *innovation trigger*, *peak of inflated expectations*, and *trough of disillusionment* as a Gaussian (Bell-shaped) curve. This function is the derivative of the logistic growth function [Eq. (1)].

3 $$Q\left( t \right) = \frac{{{\text{d}}R\left( t \right)}}{{{\text{d}}t}} = \frac{{abke^{{b\left( {t - t_{0} } \right)}} }}{{\left[ {a + e^{{b\left( {t - t_{0} } \right)}} } \right]^{2} }}$$

In contrast, S(t′) shows the *slope of enlightenment* and *plateau of productivity*. This is a modified logistic growth function.

4 $$S\left( {t^\prime } \right) = jR\left( {t^\prime } \right) = \frac{jk}{{1 + ae^{{ - b\left( {t^\prime - t_{0} } \right)}} }},$$

where *j* is the proportionality constant and *t*′ = *t* − t*. t* is the modifier of the delay to reach the *plateau of productivity.*

The proposed approach to estimate the achievement of each TRL stage involved fitting technology publications to the logistic growth behavior for scientific/engineering papers and patents, and to the hype-type behavior for news records. The standard error of the regression (S) was used as the estimator of goodness of fit. It is relevant to mention that the *S* value was chosen and not the coefficient of determination (R^2^)—which is frequently used— because research shows that the *R*^2^ is invalid for non-linear regression models (Spiess and Neumeyer 2010). The S value is defined as “the average distance that the observed values fall from the regression line” (Frost 2014) and it is measured in the units of the response variable (records). The lower the value of S, the better the model describes the response. The Marquardt-Levenberg algorithm (Marquardt 1963) was used in statistical software *Minitab 18™* to fit the logistic growth and hype-type evolution curves, as well as to obtain the S value.

**Fig. 4:**
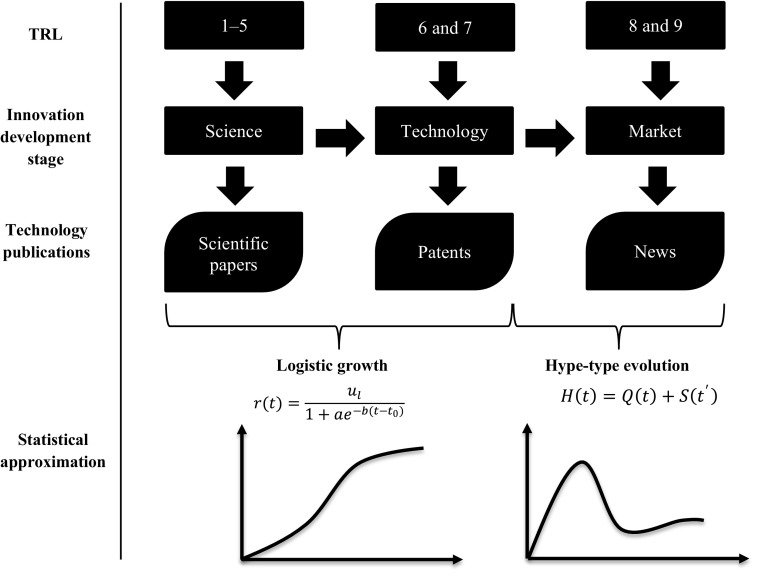
TRL estimation through technology publications

Figure 4 illustrates the approach proposed for estimating the TRLs from technology publications.

**Fig. 5:**
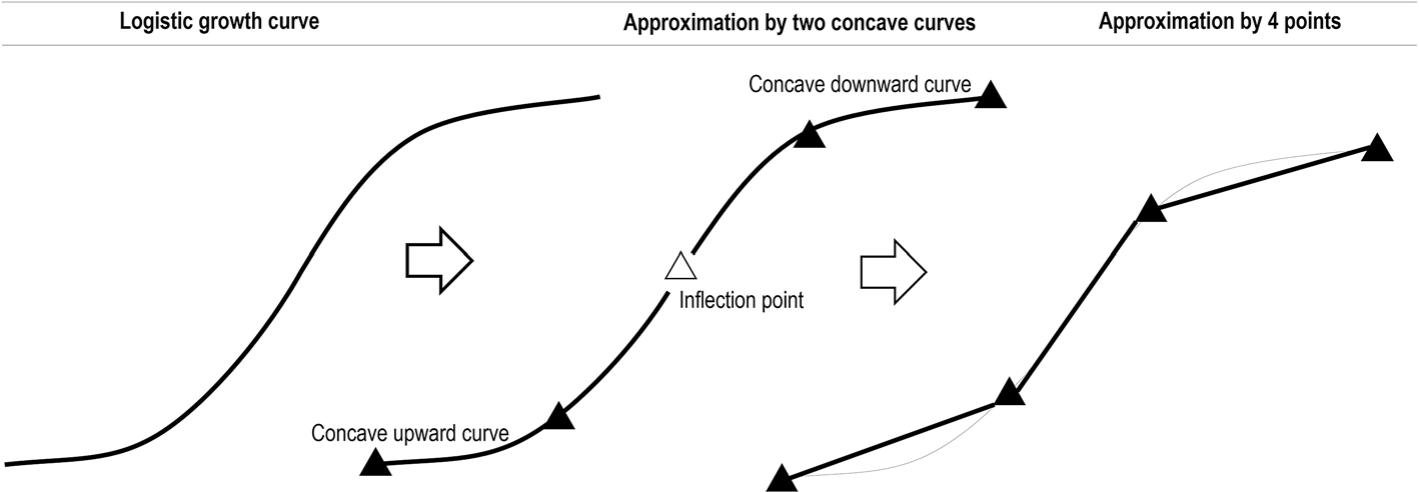
Minimum number of points to approximate the logistic growth curve

To proceed with the proposed method, there are initial conditions to be met with regard to the minimum number of records required to mathematically model the behavior of logistic growth and hype-type evolution. For logistic growth behavior, it can be graphically approximated as the superposition of two concave curves (upward and downward). The minimum number of periods of records can be considered as the minimum number of points necessary to depict this behavior, which is four. Figure 5 shows this approximation.

**Fig. 6:**
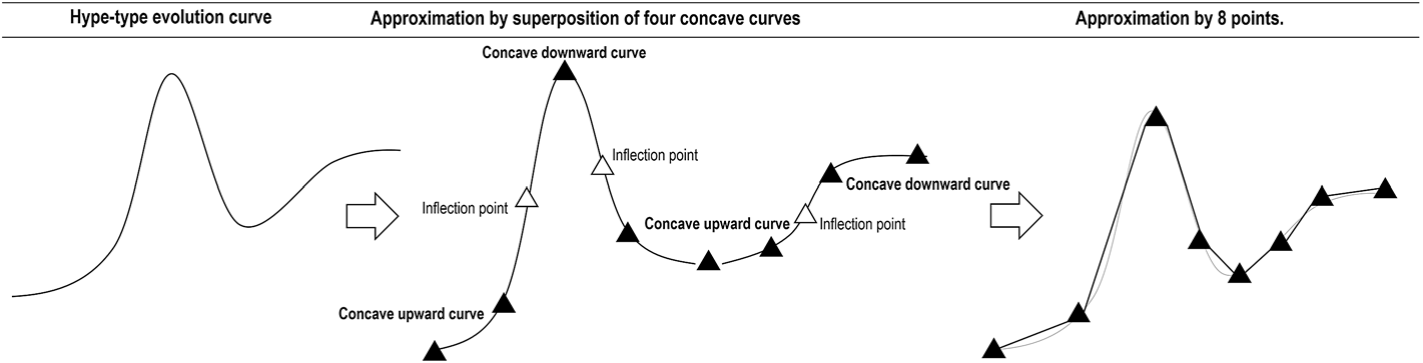
Minimum number of points to approximate the hype-type evolution curve

Conversely, the hype-type evolution can be perceived as the superposition of a Gaussian and a logistic curve, which in turn, can be graphically approximated as the superposition of three concave curves (upward-downward-upward) for the Gaussian curve, and as the superposition of two concave curves (upward-downward) for the logistic curve. The minimum number of periods of records can be considered as the minimum number of points necessary to depict this behavior, which is eight. Figure 6 shows this approximation.

The following step involved adjusting the acceptance threshold for S value (ATS) to determine whether a technology has passed the different database stages. To estimate the ATS, we used the 10 mature technologies listed in Table 3 and obtained the publication frequencies of records for the databases listed in Table 4. The search query was adjusted for the syntax of each database and covered a span from the earliest possible year per database until 2016. Each search query can be found in Appendix of Electronic Supplementary Material 1. Then, we normalized the retrieved records and fitted the logistic growth and hype-type evolution functions to get a standardized S value; where the *k* was obtained by selecting the maximum record value for all years of the sample and the *a*, *b*, *j*, and t^*^ coefficients were obtained via Marquardt-Levenberg algorithm executed in Minitab 18™ with a starting value of 0.5. The statistical summary for the fitting of each technology in each database can be found in Appendix of Electronic Supplementary Material 2. An upper bound of 95% prediction intervals was extracted to determine a more realistic ATS value. The prediction intervals are a range of values associated with a random variable yet to be observed (Hyndman 2013). The estimation of the upper bound of 95% prediction intervals was executed in Minitab 18™. A test for detecting outliers (Grubb’s test) was executed a priori to avoid extreme values that could bias the assessment. The statistical summaries for the outliers test and prediction intervals can be found in Appendices 3 and 4, respectively. The resulting S values are enlisted in Table 4.

**Table 4:** The S value for each TLC stage of the 10 mature technologies

Technologies	Databases			
	Logistic growth fit			Hype-type evolution fit
	Science Citation Index™ (TRL 3)	INSPEC™ (TRL 4–5)	Patseer™ (TRLs 6 and 7)	Factiva™ (TRL 8 and 9)
	S value			
Cloud computing	5	7	8	3
Datamining	9	9	13	3
Location-aware Technology	21*	13	18	9
Microelectromechanical systems	5	5	12	11
Organic light emitting diode	4	8	10	6
Radio-frequency identification	6	12	18	20
Smartphone	4	1	9	14
Speech recognition	8	9	13	26
Text to speech	13	15	16	24
Wireless local area network	10	6	14	24
S value average	8	9	13	14
ATS (Upper bound 95% prediction interval)	15	18	21	35

*Outlier. Detected in Minitab 18™ through the Grubb’s Test. This datum was removed to diminish bias in the prediction interval used to define the ATS

This section set the theoretical foundations for understanding how bibliometric trends can be used to estimate the level of technological maturity. The following section will describe the BIMATEM. Next, it will be applied to the seven AM technologies officially recognized by the ASTM.

## Bimatem

**Fig. 7:**
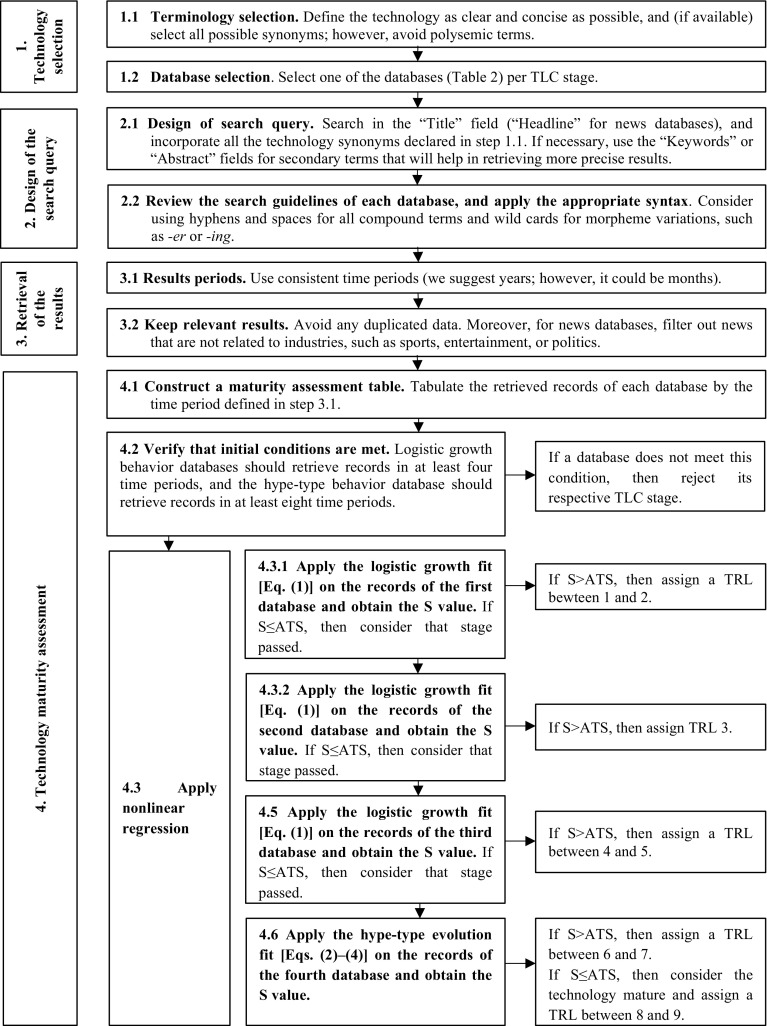
Schematic showing the workflow of the Bibliometric Method for Assessing Technological Maturity (BIMATEM)

The BIMATEM develops the methodology to estimate the TRL from publication sources. Figure 7 shows a schematic of its workflow.

### Technology selection

Step 1 of the method consists in knowing the specific technology to be assessed. It is crucial to cautiously define the scope, as some technologies tend to open into further sub-technologies. The following sub-steps state the initial guidelines to be followed on the BIMATEM.

#### Terminology selection

The technology terminology must be fully considered. It should be defined as clear and concise as possible. If possible, all synonyms should be selected. However, any polysemic (having more than one meaning) synonym should be discarded. To achieve this, an extensive literature revision is required. In addition, expert validation may work as well.

#### Database selection

Select one database per TLC stage from Table 2. Be careful to select proper databases for the technology under assessment. This becomes especially important with medical and biological sciences that tend to be indexed in separate databases.

Here ends step 1 of the BIMATEM.

### Search query

Step 2 of BIMATEM consists in constructing the search query for further results retrieval. This is one of the most delicate steps along the method. A wrong query retrieves erroneous results and produces a further sloppy assessment. The following subsections outlines some advises for a sound design of a search query.

#### Design of search query

Search queries work as chains of terms where several conditions should be met to retrieve different results. These chains are linked with search query operators. These terms are Boolean (logic) operators such as “OR” and “AND”; and proximity, such as “NEAR/#”. Additionally, there are exclusion operators such as “NOT”.

Since the BIMATEM aims at retrieving the most relevant records on technology it is necessary to deploy the search query in the “Title” fields (“Headline” for news databases) and incorporate all the technology synonyms declared in “Terminology selection” section. If necessary, use the “Keywords” or “Abstract” fields for secondary terms that aids to retrieve more precise results.

In addition, it should be noted that terms might apply differently throughout databases. For instance, “additive manufacturing” is the official term for technologies that join materials (typically layer-by-layer) to create objects from a 3D model (ASTM International 2015). However, most news records recognize it simply by the term “3D printing.” The search queries should be tested, manually checked and refined iteratively in every database to assure a relevant corpus of results. A sound query will produce clean results. (“Results relevance” section delves deeper into this matter).

Regarding patent databases, it is common to use the International Patent Classification (IPC) to filter specific technological developments. Nonetheless, we do not recommend using IPCs when analyzing emerging technologies (such as AM) because, at early stages, there are no specific IPCs for such technologies. For instance, the B33Y classification code (for additive manufacturing technologies) was created in 2015. Prior to that, AM inventions were classified through different codes. Narrowing the patent query to IPCs (in this case) would lead to a highly reduced corpus of results.

#### Revision of database guidelines and syntax

A comprehension of guidelines and syntax of the selected databases is crucial. Especially, as some databases offer different search operators, fields and rules. Adaptation to each database guidelines is necessary.

Many databases favor a more effective search query construction by allowing wildcards. These are characters that enable more than one possible interpretation. For instance, the two most common wildcards are an asterisk (*) and a hyphen (-). The former means that any number of characters (including zero) may take that place. The latter implies that either a space or a hyphen may take that place. Therefore, the term “3D print*” matches “3D printed”, “3D printing” and “3D printers”, etc.; while “3D-print” matches “3D-print” and “3D print”. Moreover, some databases cover regular plurals and inflected forms. Knowing this kind of information is imperative for an effective retrieval, since databases have a term limit for search queries. For instance, WoS allows a maximum of 6000 terms in search query and 49 Boolean operators (Clarivate Analytics 2017b).

### Retrieval of results

Step 3 of BIMATEM consists in downloading the search query results. The following sub-steps describe the guidelines for an assertive results retrieval.

#### Results periods

The periods of results retrieval should be consistent. The most appropriate period is years. However, shorter spans might be an alternative—such as months—for fast pacing technologies.

#### Results relevance

It is critical to assure a clean results retrieval. To attain this, a sound search query must be first defined, and its results must be reviewed to guarantee the soundness of the query. Furthermore, since we are counting publication frequencies, we must assure that records are not duplicated. Most scientific and engineering databases do this by default. However, this can get especially tricky in patent databases since a single patent can be applied more than once in different offices. To remove duplicated patents, we suggest filtering results by “simple patent families,” i.e., “a collection of patent documents that cover a single invention” (European Patent Office 2017). In addition, patents have application and publication dates. We retrieve the results from application dates since most AM technologies under analysis are emerging and publication dates take several months to appear. For instance, under the Patent Cooperation Treaty scheme, it takes 18 months from the first application date (WIPO 2015) for publication. It is also pertinent to notice that some patent databases gather *design patents* legally protecting industrial designs (WIPO 2017a) and *utility models* (also known as short-term patents), which are minor technological improvements on existing products (WIPO 2017b). For our research purposes, it is not necessary to include *design patents* and *utility models* since they do not represent the technical knowledge required to track the innovation development at this stage.

Most databases keep relevant results pertaining to the state of knowledge described in their information page. However, this is usually not the case of news databases that tend to index many different types of news. It is important to keep records related to industry. For *Factiva™*, these are covered under the filter “Corporate/Industrial News”. Appendix of Electronic Supplementary Material 1 shows important restrictions to keep in consideration when retrieving records from *Factiva™*.

Regarding news databases, it is important to keep records related to industry. For *Factiva™*, these are covered under the filter “Corporate/Industrial News.” Table A1.3.1 in Appendix of Electronic Supplementary Material 1 shows important restrictions to be considered when retrieving records from *Factiva™*. The interpretation of results is performed in the following section.

Here ends step 3 of the BIMATEM.

### Technology maturity assessment

Step 4 of the method evaluates mathematically the retrieved records and assigns a TRL to the technology under assessment. The following sub-steps describe the guidelines to quantify the technological maturity.

#### Construct a maturity assessment table

This step consists in tabulating the retrieved records of every database that complied with the conditions stated in “Construct a maturity assessment table” section. Use the time period defined in “Results periods” section. The maturity assessment table should contain the number of periods to verify compliance of initial conditions as well as records per year. Table 5 shows the maturity assessment table to be constructed in statistical software. This research uses *Minitab 18™*.

**Table 5:** Maturity assessment table

YEAR	YEAR-INITIAL_YEAR	RECORDS	NORMALIZED RECORDS
Year_1	(Year_1-Year_1)	Records_in_Year_1	(Records_in_Year_1/ MAX(RECORDS)
Year…	(Year…-Year_1)	Records_in_Year…	(Records_in_Year…/ MAX(RECORDS)
Year_n	(Year_n-Year_1)	Records_in_Year_n	(Records_in_Year_n/ MAX(RECORDS)

The YEAR and RECORDS columns are obtained directly from results retrieval

The YEAR-INITIAL_YEAR column is obtained by subtracting the value of the initial year to every year and the YEAR-INITIAL_YEAR column is filled by dividing each record over the maximum record value for all years

#### Verify that initial conditions are met

The conditions stated in “Technology selection” section and “Search query” section must be complied. Logistic growth behavior databases should retrieve records in at least 4 periods and the hype-type behavior database should retrieve records in at least 8 periods. If a database retrieves fewer records, then the respective stage should be rejected.

#### Apply non-linear regression to each database

This sub-step consists in applying non-linear regression to the maturity assessment tables constructed in “Construct a maturity assessment table” section. Logistic growth regression (Eq. 1) should be applied on records of basic research, applied research and development databases, whereas hype-type regression (Eq. 2) should be applied on records of news databases. Databases that did not comply the initial conditions of “Design of the search query” section should not be considered.

The *Levenberg*-*Marquardt* nonlinear regression algorithm should be executed on statistical software. Nonlinear regression algorithms require starting values. Since the data are adjusted on a scale from 0 to 1, the constant *k* will be fixed to the maximum value (1) and the initial values *a* and *b* for logistic growth fitting, and *a, b, j* and t* for hype-type evolution may be all started at 0.5.

**Fig. 8:**
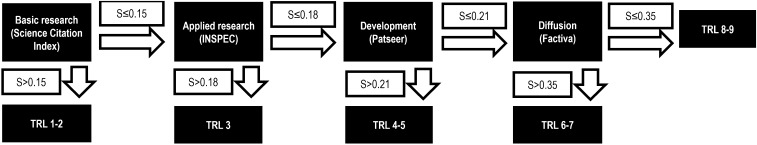
TRL assignation from BIMATEM

Once the nonlinear regression is executed, the estimation of the TRL may be executed by comparing the obtained S value versus the ATS. Figure 8 presents the algorithm to obtain the TRLs in the last step of BIMATEM. Here ends BIMATEM.

The next section discusses the application of this method to AM technologies.

## Case of application: AM technologies

AM, commonly known as three-dimensional (3D) printing, is a transformative technology wherein a 3D computer-aided design system can fabricate objects layer by layer by joining materials (Wohlers and Caffrey 2015). It is a promising technology that has the potential to substantially simplify the process of producing three-dimensional objects (Gibson et al. 2010).

AM is a significant stepping stone in the global shift toward mass customization. Supply chains are likely to shrink for many products that are in demand (Campbell et al. 2011), logistic and energy costs are expected to be overthrown (Rifkin 2012), and a disruptive transformation in business model is estimated to occur across different industries (Shanler and Basiliere 2017).

AM has resulted in an appealing case of study for assessing technological maturity because it has revolutionizing potential. It is unfolding into further promising technologies, such as *nanoscale printing* (Shanler and Basiliere 2017) or *bioprinting* (Rodriguez-Salvador et al. 2017). The BIMATEM proposed herein was applied to AM technologies. Each step of the method is described in the following subsections to provide relevant insights for technology managers and policymakers.

### Terminology selection

AM technologies are officially classified in a set of seven unique processes with varying characteristics (ASTM International 2015). These technologies are defined in Table 6, along with their most concise synonyms or their most remarkable processes.

**Table 6:** The seven additive manufacturing (AM) technologies, officially recognized by the American Society for Testing and Materials [ASTM; ASTM International (2015)], to be tested using the Bibliometric Method for Assessing Technological Maturity (BIMATEM)

Technology	Synonyms/most remarkable processes	Definition (Shanler and Basiliere 2017)
Binder jetting	Voxeljet	Liquid bonding agent is selectively deposited to join powder materials
Directed energy deposition	Laser cladding, laser-engineered net shaping, laser-based metal deposition, laser freeform fabrication, laser direct casting, laser consolidation, directed light fabrication, and direct metal deposition	Thermal energy is used to fuse materials by melting as the material is being deposited
Material extrusion	Fused filament fabrication, fused deposition/layer modeling, and plastic jet printing	Material is selectively dispensed through a nozzle or orifice
Material jetting	Multijet modeling, thermojet, and inkjet printing	Droplets of build material are selectively deposited
Powder bed fusion	Direct metal laser sintering, selective laser melting/sintering, and electron beam melting	Thermal energy selectively fuses regions of powder bed
Sheet lamination	Ultrasonic additive manufacturing, ultrasonic consolidation, and lamination object manufacturing	Sheets of material are bonded to form an object
Vat photopolymerization	Stereolithography, SLA, SL, and thin-film photopolymerization	Liquid photopolymer in a vat is selectively cured by light-activated polymerization

The next step toward assessing the maturity level from the BIMATEM involved converting each AM technology concept into a search query. This is addressed in the following section.

### Design of the search query

Table 7 lists the general search queries of the previously defined technologies. The operator “OR” retrieves records containing any terms within the query, “AND” recovers records that contain all the terms in the query, and “NEAR/#” retrieves records whose terms are joined at a maximum distance of # words. In addition, the syntax includes two wildcards: an asterisk (*) and a hyphen (-). The former means that any number of characters (including zero) may take that place. The latter implies that either a space or a hyphen may take that place. Therefore, the term “3D print*” matches “3D printed,” “3D printing,” and “3D printers,” while “3D-print” matches “3D-print” and “3D print.” Moreover, the terms are only shown in singular, although plural—and further syntax—variations were adjusted for each database requirements (refer to Appendix of Electronic Supplementary Material 5 to review the search query adaptations to each database in more detail). The query was launched from the earliest possible date per database until 2016.

**Table 7:** Search query for AM technologies

Technology	Search query
Binder jetting	Title: (*Binder*-jet* OR *Voxeljet*) Abstract/Keywords: ((*3D* OR *3*-*Dimensional* OR *three*-*D* OR *three*-*dimensional*) NEAR/1 (Print*)) OR (Additive manufactur*)
Directed energy deposition	Title: (Direct* energy deposition OR Laser clad* OR *Laser*-*engineered net shaping* OR ((*Laser* OR *Direct*) NEAR/1 (*Metal*-*deposition*)) OR *Laser freeform*-*fabrication* OR *Laser direct*-*casting* OR *Laser*-*consolidation* OR ((Direct*) NEAR/1 *Light fabrication*)) Abstract/Keywords: ((*3D* OR *3*-*Dimensional* OR *three*-*D* OR *three*-*dimensional*) NEAR/1 (Print*)) OR (Additive manufactur*)
Material extrusion	Title: (*Material extrusion* OR Fuse* filament-fabricat* OR Fuse* deposition-model* OR Fuse* layer*model* OR *Plastic jet*-print*) Abstract/Keywords: ((*3D* OR *3*-*Dimensional* OR *three*-*D* OR *three*-*dimensional*) NEAR/1 (Print*)) OR (Additive manufactur*)
Material jetting	Title: (Material jet* OR (*Multijet* OR *Multi*-*jet)* NEAR/1 model*) OR *Thermojet* OR (*Inkjet* OR *Ink*-*jet)* NEAR/1 print*) Abstract/Keywords: ((*3D* OR *3*-*Dimensional* OR *three*-*D* OR *three*-*dimensional*) NEAR/1 (Print*)) OR (Additive manufactur*)
Powder bed fusion	Title: (*Powder bed fusion* OR *Direct*-metal laser sinter* OR (*Selective laser* OR *Electron beam*) NEAR/1 (Melt* OR Sinter*)) Abstract/Keywords: ((*3D* OR *3*-*Dimensional* OR *three*-*D* OR *three*-*dimensional*) NEAR/1 (Print*)) OR (Additive manufactur*)
Sheet lamination	Title: (Sheet laminat* OR (*Ultrasonic* NEAR/1 (Consolidat* OR Additive manufactur*)) OR Lamination object manufactur*) Abstract/Keywords: ((*3D* OR *3*-*Dimensional* OR *three*-*D* OR *three*-*dimensional*) NEAR/1 (Print*)) OR (Additive manufactur*)
Vat photopolymerization	Title: (Vat photopolymerizat* OR Stereolithograph* OR *SLA* OR *Thin*-film photopolymerizat*) Abstract/Keywords: ((*3D* OR *3*-*Dimensional* OR *three*-*D* OR *three*-*dimensional*) NEAR/1 (Print*)) OR (Additive manufactur*)

The next step after defining the search query is to retrieve the records. The following section gives guidelines for optimal data retrieval.

### Retrieval of the results

Once the search query is launched, the following points must be taken into consideration.

#### Results periods

Because the finest level where the chosen databases are capable of filtering results is years, this is the period considered to be used in this application of BIMATEM.

#### Results relevance

Following the recommendations declared in “Results relevance” section, the results from every database were reviewed to detect any undesired records. Nonetheless, the query designed in “Design of the search query” section was sufficiently sound and did not retrieve noisy records. Regarding the deduplication process in patents, they were all filtered by patent family (as indicated in Table A1.2 in Appendix of Electronic Supplementary Material 1). With regards to news records, they were kept relevant by filtering only “Corporate/Industrial News” (as indicated in Table A1.3 in Appendix of Electronic Supplementary Material 1).

### Technology maturity assessment

The last step of BIMATEM consists in creating maturity assessment tables and applying logistic growth/hype-type regression to assign a TRL to each AM technology. The statistical software used in this study was *Minitab 18™.* Appendix of Electronic Supplementary Material 6 contains the maturity assessment tables for every AM technology, as well as their according curve fitting. S values were extracted from each regression; those that exceeded the ATS of their respective database were assigned to the according TRLs shown in Table 4. The BIMATEM results applied to AM technologies are summarized in Table 8.

**Table 8:** BIMATEM results for AM technologies. Check marks represent the compliance of conditions, and cross marks imply the failure of conditions. If a given database contains at least one cross mark, then that TRL stage is not passed

Technology	Database				
	Logistic growth fit			Hype-type evolution fit	
	Initial conditions: YWR≥4			Initial conditions YWR≥8	
	Science Citation Index™ (TRL3) ATS≤15	*INSPEC™* (TRLs 4-5) ATS≤18	*Patseer™* (TRLs 6-7) ATS≤21	*Factiva™* (TRLs 8-9) ATS≤35	Results
Binder Jetting	YWR < 4 × S=NA ×				TRL 1–2
Directed energy deposition	YWR = 12✓S = 9✓	YWR = 10✓ S = 9✓	YWR < 4 × S = NA✓		TRL 4–5
Material extrusion	YWR = 5✓ S = 10✓	YWR = 5✓ S = 14✓	YWR = 6✓ S = 8✓	YWR < 8 × S = NA ×	TRL 6–7
Material jetting	YWR = 12✓ S = 10✓	YWR = 14✓ S = 12✓	YWR = 11✓ S = 19✓	YWR < 8 × S = NA ×	TRL 6–7
Powder bed fusion	YWR = 10✓ S = 8✓	YWR = 10✓ S = 8✓	YWR = 4✓ S = 1✓	YWR < 8 × S = NA ×	TRL 6–7
Sheet lamination	YWR = 10✓ S = 30 ×				TRL 1–2
Vat photopolymerization	YWR = 11✓ S = 11✓	YWR = 9✓ S = 12✓	YWR = 6✓ S = 6✓	YWR < 8 × S = NA ×	TRL 6–7

S = Standard error of the regression

ATS = Acceptance threshold for S

YWR = Years with records

The following section discusses the results, benefits, setbacks, and implications concerning the AM maturity assessment results provided by the BIMATEM.

## Results and discussion

The BIMATEM assigned a TRL to the seven AM technologies (ASTM International 2015). The effectiveness of the assessment mostly relies on the accuracy of the search query and the record completeness of the database collection.

Furthermore, emerging technologies may require time to reach consensus on a given technological concept. For instance, Charles Hull filed the first patent regarding AM in 1984, which coined the term “stereolithography” in its title (Hull 1984). However, the terms “AM” or “3D printing” were not yet used. Hence, that record does not appear in vat photopolymerization search query retrieval. Standardizing technology terms is an indicator of technological maturity progress.

Binder jetting was the least mature AM technology (TRL 1-2), as it did not gather the minimum number of years of publication established in the initial conditions in the basic research (*Science Citation Index™)* database. It has been recognized as an immature technology that requires improvement in accuracy and surface finish (Gibson et al. 2010).

Sheet lamination was another technology that obtained a TRL 1-2 in the BIMATEM results. It did not display a logistic growth fit behavior, as it had an S value (30) beyond the ATS (15). It is considered as a fringe of the AM process (Gibson et al. 2010) that awaits improvements in material, bonding, and supporting methods as well as sheet placement.

Directed energy deposition was classified as TRL 4-5. It successfully passed the *Science Citation Index™* and *INSPEC™* stages. However, it did not meet the initial conditions of having at least four years of published records in *Patseer™*. It has been previously remarked for its “limited success in the AM market” (Wohlers and Caffrey 2015). It is an AM technology mainly suited for repair and feature addition. Among its biggest limitations are poor resolution surface finish, as well as low build speed.

The remaining technologies were classified as TRL 6-7. They passed the first three database stages but failed in the news database (*Factiva*™), where they did not meet the initial condition of gathering at least eight years of published records for the hype-type evolution fitting.

According to the US GAO (US Government Accountability Office 2016), technologies that have reached this level are considered sufficiently mature to be integrated into product development.

Material jetting has been used in the medical and aeronautic industry (Shanler and Basiliere 2017). It still needs to improve resolution accuracy, as well as the limited choice of materials where it can be printed (Gibson et al. 2010). In contrast, vat photopolymerization does not face resolution setbacks. However, the use of photopolymers restricts its application because they do not offer good strength or durability (Gibson et al. 2010). Among the biggest challenges faced by this technology is the development of new raw materials and integration of post-printing processes (Shanler and Basiliere 2017).

Material extrusion and powder bed fusion are probably the most promising AM technologies currently. Material extrusion has been the most commercially exploited AM technique in the market (Gibson et al. 2010). However, important challenges, such as the printing speed, material density, and accuracy, are yet to be overcome (Gibson et al. 2010). On the other hand, powder bed fusion has been successfully incorporated in aeronautical and medical industry for its near-net-shape production (Wohlers and Caffrey 2015).

The BIMATEM has allowed us to assess the technological maturity of the seven official AM technologies along the TRL scale. Its effectiveness relies on its bibliometric nature because it leaves aside experts’ assessment. It is a repeatable, reliable and semi-automated method to obtain the TRL. Its results are consistent with findings of similar reports regarding maturity of AM technologies. Hague et al (2016) positioned electron beam melting (a form of powder bed fusion) among the most mature AM technologies with a TRL 7-8. They also considered fused deposition modelling as TRL 4-6, and material jetting as TRL 2-3. Considered AM technologies as a whole, most processes have passed the basic research stage and are awaiting exploitation of their applications, averaging to TRL 4 (Hoiss et al. 2014).

Maturity analysis of AM technologies is often assessed through various perspectives. For instance, from a materials perspective, plastics are considered mature when printed for prototypes purposes (no mechanical resistance) (Wohlers and Caffrey 2015). Regarding materials with good engineering properties, metals surpass the others, where Ni-based superalloys, Co-Cr alloys, Ti-based alloys, stainless steels and tool steels are the most mature at TRL 7-9 (Gorsse et al. 2017). It is no coincidence that these materials are mostly used in powder bed fusion processes.

Another perspective often considered is the industries where specific AM technologies are being utilized. Several reports (Wohlers and Caffrey 2015; Campbell et al. 2011; Shanler and Basiliere 2017) agree that industries where AM has been predominately developed are consumer goods for product prototyping (mostly through material extrusion); the medical industry, where techniques such as material jetting (mostly through polyjet printing) have been used to print models of body parts, as well as powder bed fusion, where prosthetics are being developed and produced. Powder bed fusion has also made a great impact on aeronautic industries, where it has been used to produce aircraft components with complex geometries requiring high mechanical properties, such as rear bearing turbine supports.

After a BIMATEM application on AM technologies, a revision of its technological maturity findings and a discussion of its implications, the final section will summarize the BIMATEM development and findings as well as its setbacks and future research.

## Conclusions

William Nolte (2008) stated that “evaluating technology maturity is a far more complex subject than it appears to be.” It is an issue that has been dealt with for decades. It aims to assess the risk level that accompanies new technologies.

For years, the TRL has been regarded as an effective approach for quantitatively assessing technological maturity. Nevertheless, it is mostly determined via expert surveys, which implies the risk of personal bias. Herein, BIMATEM was developed as an approach to obtain a TRL from the science, technology, and news records. This diminishes the level of bias in the analysis and avoids the costs and drawbacks associated with assessing technological maturity through expert opinions.

The bibliometric nature of the BIMATEM offers reliability and objectivity because it is based on the statistical behavior of published records. The determination of TRLs 1–7, the most important step for decision-making purposes (US Government Accountability Office 2016), is based on fitting the records to the logistic growth function, a proven statistical behavior of records of mature technologies (Wong and Goh 2010), while the estimation of the TRL between 8 and 9 is achieved by fitting the hype-type evolution curves to the news records, a novel finding of this study.

The BIMATEM can be used to assess the maturity extent through the TRL of any technology that has published records in scientific, technological, and news databases. Its implications go beyond monitoring purposes as it can be employed in further technological planning techniques, such as roadmapping, competitive intelligence, or foresight. It can be systematically implemented in the planning agenda among organizations and used for benchmarking purposes.

The BIMATEM was tested on the seven AM technologies officially recognized by the ASTM (ASTM International 2015). The obtained results were consistent to the challenges that those technologies face currently.

The TRL offers solid insights for technology managers. Incorporating the BIMATEM into planning activities within organizations would enable them to assess the risk associated with acquiring or developing new technologies. In addition, the method can be integrated with other approaches for tracking technology development. For instance, it can be used for competitive technology intelligence purposes to benchmark technological sectors or for foresight analysis to provide technological maturity insights for industrial sectors and territories.

BIMATEM assumes a linear innovation pathway. Thus, it requires technologies that have clearly left evidence in every stage of the linear innovation model (i.e., basic research, applied research, product development, and social impact).

Another characteristic of our method is that it deploys the TRL in five chunks (TRL between 1 and 2, TRL 3, TRL between 4 and 5, TRL between 6 and 7, and TRL between 8 and 9) rather than the nine-level scale. This may be a setback for designers or technicians directly involved in technology development. However, at the strategic level, this can be considered an advantage as a reduced scale may facilitate decision-making for technology managers.

One limitation of our method is that a technology can never achieve a TRL 3 under four time units (years for our case of study). Shorter time units, such as months, can be used instead for fast paced technologies. Another limitation of BIMATEM is that it relies on records that lag to appear: scientific papers and patents may require a period of months or even years from initial submission until they are finally published. This may result in inaccurate publication counts for fast-paced technologies. To fill this gap, future lines of research can work on testing this model with further bibliometric and information sources, such as videos or social media interaction. Additionally, *design patents* or *utility models* might be implemented to trace technology maturation at a more granular level.

## Electronic supplementary material

Below is the link to the electronic supplementary material.

Supplementary material 1 (DOCX 117 kb)
