# Supplementary material for: A bibliometric method for assessing technological maturity: the case of additive manufacturing
Source: Scientometrics. 2018 Nov 1;117(3):1425–52. doi: 10.1007/s11192-018-2941-1 (PMC6267247; doi:10.1007/s11192-018-2941-1)
Supplement: Supplementary file 1 — Supplementary material 1 (DOCX 117 kb) [file 11192_2018_2941_MOESM1_ESM.docx]

**Appendix 1.** Search queries for 10 mature technologies

**Table A1.1** Search queries for 10 mature technologies on *Science Citation Index™* and *INSPEC™* databases

| Technology | Search query |
| --- | --- |
| Cloud computing | TI: Cloud-computing |
| Datamining | TI=(Datamining or Textmining or Data-mining or Text-mining) |
| Location-Aware Technology | TI=(Location-aware* OR Location-intelligen*) |
| Microelectromechanical systems | TI=(("MEMS" OR (Microelectromechanic* OR Micro-electro-mechanic* OR Microelectronic* OR Microelectromechanic*) NEAR/0 (System)) NOT ("Medication Event Monitor* System" OR "Medication Management Systems")) |
| Organic light emitting diode | TI= (("Organic light emitt*") NEAR/0 (Device OR Diode) OR OLED) |
| Radio Frequency Identification (RFID) | TI= ((RFID OR (Radio-frequency NEAR/1 identif*))) |
| Smartphone | TI=("Smart phone" OR smartphone) |
| Speech recognition | TI=((Speech) NEAR/0 (Recognition OR to-text)) |
| Text to speech | TI= ("Text-to-speech" OR "Speech synthesis" OR "Text-to-voice") |
| Wireless Local Area Network | TI=("Wireless LAN" OR WLAN OR WiFi OR "IEEE 802.11" OR "IEEE STD 802.11" OR "IEEE 802 standard") |

**Table A1.2** Search queries for 10 mature technologies on *Patseer™*

| Technology | Search query |
| --- | --- |
| Cloud computing | T: Cloud-computing |
| Datamining | T:(Datamining or Textmining or Data-mining or Text-mining) |
| Location-Aware Technology | T:(Location-aware* OR Location-intelligen*) |
| Microelectromechanical systems | T:(("MEMS" OR (Microelectromechanic* OR Micro-electro-mechanic* OR Microelectronic* OR Microelectromechanic*) Wd0 (System)) NOT (Medication Event Monitor* System OR Medication Management System)) AND PTYP:("Patent" OR "Application") |
| Organic light emitting diode | T:((Organic light emitt*) wd0 (Device OR Diode) OR OLED) |
| Radio Frequency Identification (RFID) | T:(RFID OR (Radio-frequency wd1 identif*)) |
| Smartphone | T: smart-phone OR smartphone |
| Speech recognition | T: ((Speech) wd0 (Recognition OR to-text)) |
| Text to speech | T:(Text-to-speech OR Speech synthesis OR Text-to-voice) |
| Wireless Local Area Network | T:(Wireless LAN OR WLAN OR WiFi OR "IEEE 802.11" OR "IEEE STD 802.11" OR "IEEE 802 standard") |

**Table A1.3** Search queries for 10 mature technologies on *Factiva™*

| Technology | Search query |
| --- | --- |
| Cloud computing | (Cloud computing) |
| Datamining | (Datamining or Textmining or Data-mining or Text-mining) |
| Location-Aware Technology | (Location-aware* OR Location-intelligen*) |
| Microelectromechanical systems | (MEMS OR (Microelectromechanic* OR Micro-electro-mechanic* OR Microelectronic* OR Microelectromechanic*) NEAR1 (System)) |
| Organic light emitting diode | ((Organic light emitt*) NEAR1 (Device OR Diode) OR OLED) |
| Radio Frequency Identification (RFID) | (RFID OR Radio-frequency identificat*) |
| Smartphone | (Smartphone OR smart-phone) |
| Speech recognition | ((Speech) NEAR1 (Recognition OR to-text)) |
| Text to speech | (Text-to-speech OR Speech-synthesis OR Text-to-voice) |
| Wireless Local Area Network | (Wireless LAN OR WLAN OR WiFi OR IEEE 802.11 OR IEEE STD 802.1 OR IEEE 802 standard) |

**Table A1.3.1** Parameters considered for search queries on *Factiva™*

| Search fields: | Headline |
| --- | --- |
| Date: | All Dates |
| Exclude: | Republished news. Recurring pricing and market data. Obituaries, sports, calendars… |
| Source: | All Sources |
| Author: | All Authors |
| Company: | All Companies |
| Subject: | Corporate/Industrial News NOT (Commodity/Financial Market News OR Content Types OR Economic News Not Political/General News OR Selection of Top Stories/Trends/Analysis OR Sports) |
| Industry: | All Industries |
| Region: | All Regions |
| Language: | English |

**Appendix 2.** Curve fitting of mature technologies

**A2.1 Cloud computing**

**Table A2.1.1**: Retrieved records of cloud computing from Science Citation Index™

| Year | Year-Initial year | Records | Normalized records |
| --- | --- | --- | --- |
| 2007 | 0 | 1 | 0.00286533 |
| 2008 | 1 | 3 | 0.008595989 |
| 2009 | 2 | 33 | 0.094555874 |
| 2010 | 3 | 65 | 0.186246418 |
| 2011 | 4 | 116 | 0.332378223 |
| 2012 | 5 | 152 | 0.435530086 |
| 2013 | 6 | 265 | 0.759312321 |
| 2014 | 7 | 277 | 0.793696275 |
| 2015 | 8 | 340 | 0.974212034 |
| 2016 | 9 | 349 | 1 |

**Minitab 18™ summary for logistic growth fit of cloud computing records from *Science Citation Index™***

**Method**

| Algorithm | Marquardt |
| --- | --- |
| Max iterations | 200 |
| Tolerance | 0.00001 |

**Equation**

Cloud computing records from SCI = 1 / (1 + 64.593 * EXP(-0.83909 * 'Year-Initial_Year'))

**Summary**

| Iterations | 12 |
| --- | --- |
| Final SSE | 0.0165996 |
| DFE | 8 |
| MSE | 0.0020749 |
| S | 0.0455516 |

**Table A2.1.2**: Retrieved records of cloud computing from INSPEC™

| Year | Year-Initial year | Records | Normalized records |
| --- | --- | --- | --- |
| 2003 | 0 | 1 | 0.000672043 |
| 2008 | 5 | 30 | 0.02016129 |
| 2009 | 6 | 222 | 0.149193548 |
| 2010 | 7 | 560 | 0.376344086 |
| 2011 | 8 | 976 | 0.655913978 |
| 2012 | 9 | 1187 | 0.797715054 |
| 2013 | 10 | 1478 | 0.99327957 |
| 2014 | 11 | 1488 | 1 |
| 2015 | 12 | 1403 | 0.942876344 |
| 2016 | 13 | 1224 | 0.822580645 |

**Minitab 18™ summary for logistic growth fit of cloud computing records from *INSPEC™***

**Method**

| Algorithm | Marquardt |
| --- | --- |
| Max iterations | 200 |
| Tolerance | 0.00001 |

**Equation**

Cloud computing records from *INSPEC™* = 1 / (1 + 5163.96 * EXP(-1.1408 * 'Year-Initial_Year'))

**Summary**

| Iterations | 24 |
| --- | --- |
| Final SSE | 0.0402036 |
| DFE | 8 |
| MSE | 0.0050254 |
| S | 0.0708904 |

**Table A2.1.3**: Retrieved records of cloud computing from Patseer™

| Year | Year-Initial year | Records | Normalized records |
| --- | --- | --- | --- |
| 2008 | 0 | 15 | 0.020891365 |
| 2009 | 1 | 98 | 0.136490251 |
| 2010 | 2 | 307 | 0.427576602 |
| 2011 | 3 | 537 | 0.747910864 |
| 2012 | 4 | 667 | 0.928969359 |
| 2013 | 5 | 718 | 1 |
| 2014 | 6 | 676 | 0.941504178 |
| 2015 | 7 | 618 | 0.860724234 |
| 2016 | 8 | 619 | 0.862116992 |

**Minitab 18™ summary for logistic growth fit of cloud computing records from *Patseer™***

**Method**

| Algorithm | Marquardt |
| --- | --- |
| Max iterations | 200 |
| Tolerance | 0.00001 |

**Equation**

Cloud computing records from *Patseer™* = 1 / (1 + 25.6862 * EXP(-1.45513 * 'Year-Initial_Year'))

**Summary**

| Iterations | 11 |
| --- | --- |
| Final SSE | 0.0418069 |
| DFE | 7 |
| MSE | 0.0059724 |
| S | 0.0772814 |

**Table A2.1.4**: Retrieved records of cloud computing from Factiva™

| Year | Year-Initial year | Records | Normalized records |
| --- | --- | --- | --- |
| 2007 | 0 | 4 | 0.002435 |
| 2008 | 1 | 146 | 0.088862 |
| 2009 | 2 | 585 | 0.356056 |
| 2010 | 3 | 1183 | 0.720024 |
| 2011 | 4 | 1643 | 1 |
| 2012 | 5 | 1139 | 0.693244 |
| 2013 | 6 | 800 | 0.486914 |
| 2014 | 7 | 825 | 0.50213 |
| 2015 | 8 | 746 | 0.454047 |
| 2016 | 9 | 708 | 0.430919 |

**Minitab 18™ summary for hype-type evolution fit of cloud computing records from *Factiva™***

**Method**

| Algorithm | Marquardt |
| --- | --- |
| Max iterations | 200 |
| Tolerance | 0.0001 |

**Equation**

Cloud computing from *Factiva™* = 3539.79 * 2.09157 * 1 * EXP(2.09157 * 'Year-Initial_Year') / ((3539.79 + EXP(2.09157 * 'Year-Initial_Year')) ^ 2) + 0.479422 * 1 / (1 + 3539.79 * EXP(-2.09157 * (Year + 2.23269 - 2007)))

**Summary**

| Iterations | 24 |
| --- | --- |
| Final SSE | 0.0065373 |
| DFE | 6 |
| MSE | 0.0010895 |
| S | 0.0330082 |

**A2.2 Datamining**

**Table A2.2.1**: Retrieved records of datamining from Science Citation Index™

| Year | Year-Initial year | Records | Normalized records |
| --- | --- | --- | --- |
| 1983 | 0 | 1 | 0.002197802 |
| 1993 | 10 | 1 | 0.002197802 |
| 1994 | 11 | 4 | 0.008791209 |
| 1995 | 12 | 9 | 0.01978022 |
| 1996 | 13 | 24 | 0.052747253 |
| 1997 | 14 | 56 | 0.123076923 |
| 1998 | 15 | 77 | 0.169230769 |
| 1999 | 16 | 120 | 0.263736264 |
| 2000 | 17 | 137 | 0.301098901 |
| 2001 | 18 | 148 | 0.325274725 |
| 2002 | 19 | 198 | 0.435164835 |
| 2003 | 20 | 239 | 0.525274725 |
| 2004 | 21 | 292 | 0.641758242 |
| 2005 | 22 | 317 | 0.696703297 |
| 2006 | 23 | 329 | 0.723076923 |
| 2007 | 24 | 253 | 0.556043956 |
| 2008 | 25 | 281 | 0.617582418 |
| 2009 | 26 | 321 | 0.705494505 |
| 2010 | 27 | 315 | 0.692307692 |
| 2011 | 28 | 335 | 0.736263736 |
| 2012 | 29 | 360 | 0.791208791 |
| 2013 | 30 | 345 | 0.758241758 |
| 2014 | 31 | 371 | 0.815384615 |
| 2015 | 32 | 373 | 0.81978022 |
| 2016 | 33 | 455 | 1 |

**Minitab 18™ summary for logistic growth fit of datamining records from *Science Citation Index™***

**Method**

| Algorithm | Marquardt |
| --- | --- |
| Max iterations | 200 |
| Tolerance | 0.00001 |

**Equation**

Datamining records from SCI = 1 / (1 + 83.3134 * EXP(-0.208079 * 'Year-Initial_Year'))

**Summary**

| Iterations | 21 |
| --- | --- |
| Final SSE | 0.173865 |
| DFE | 23 |
| MSE | 0.0075593 |
| S | 0.0869444 |

**Table A2.2.2**: Retrieved records of datamining from INSPEC™

| Year | Year-Initial year | Records | Normalized records |
| --- | --- | --- | --- |
| 1993 | 0 | 2 | 0.001881468 |
| 1994 | 1 | 8 | 0.00752587 |
| 1995 | 2 | 25 | 0.023518344 |
| 1996 | 3 | 86 | 0.080903104 |
| 1997 | 4 | 157 | 0.147695202 |
| 1998 | 5 | 225 | 0.211665099 |
| 1999 | 6 | 247 | 0.232361242 |
| 2000 | 7 | 283 | 0.266227658 |
| 2001 | 8 | 309 | 0.290686736 |
| 2002 | 9 | 396 | 0.372530574 |
| 2003 | 10 | 349 | 0.328316087 |
| 2004 | 11 | 421 | 0.396048918 |
| 2005 | 12 | 496 | 0.466603951 |
| 2006 | 13 | 583 | 0.548447789 |
| 2007 | 14 | 703 | 0.661335842 |
| 2008 | 15 | 851 | 0.80056444 |
| 2009 | 16 | 1063 | 1 |
| 2010 | 17 | 973 | 0.91533396 |
| 2011 | 18 | 851 | 0.80056444 |
| 2012 | 19 | 811 | 0.762935089 |
| 2013 | 20 | 806 | 0.758231421 |
| 2014 | 21 | 875 | 0.823142051 |
| 2015 | 22 | 855 | 0.804327375 |
| 2016 | 23 | 920 | 0.865475071 |

**Minitab 18™ summary for logistic growth fit of datamining records from *INSPEC™***

**Method**

| Algorithm | Marquardt |
| --- | --- |
| Max iterations | 200 |
| Tolerance | 0.00001 |

**Equation**

Datamining records from *INSPEC™* = 1 / (1 + 17.9738 * EXP(-0.24874 * 'Year-Initial_Year'))

**Summary**

| Iterations | 15 |
| --- | --- |
| Final SSE | 0.176829 |
| DFE | 22 |
| MSE | 0.0080377 |
| S | 0.0896531 |

**Table A2.2.3**: Retrieved records of datamining from Patseer™

| Year | Year-Initial year | Records | Normalized records |
| --- | --- | --- | --- |
| 1995 | 0 | 4 | 0.019607843 |
| 1996 | 1 | 7 | 0.034313725 |
| 1997 | 2 | 10 | 0.049019608 |
| 1998 | 3 | 15 | 0.073529412 |
| 1999 | 4 | 27 | 0.132352941 |
| 2000 | 5 | 43 | 0.210784314 |
| 2001 | 6 | 53 | 0.259803922 |
| 2002 | 7 | 68 | 0.333333333 |
| 2003 | 8 | 70 | 0.343137255 |
| 2004 | 9 | 60 | 0.294117647 |
| 2005 | 10 | 63 | 0.308823529 |
| 2006 | 11 | 46 | 0.225490196 |
| 2007 | 12 | 70 | 0.343137255 |
| 2008 | 13 | 76 | 0.37254902 |
| 2009 | 14 | 56 | 0.274509804 |
| 2010 | 15 | 58 | 0.284313725 |
| 2011 | 16 | 72 | 0.352941176 |
| 2012 | 17 | 80 | 0.392156863 |
| 2013 | 18 | 116 | 0.568627451 |
| 2014 | 19 | 131 | 0.642156863 |
| 2015 | 20 | 193 | 0.946078431 |
| 2016 | 21 | 204 | 1 |

**Minitab 18™ summary for logistic growth fit of datamining records from *Patseer™***

**Method**

| Algorithm | Marquardt |
| --- | --- |
| Max iterations | 200 |
| Tolerance | 0.00001 |

**Equation**

Datamining records from *Patseer™* = 1 / (1 + 16.3542 * EXP(-0.178517 * 'Year-Initial_Year'))

**Summary**

| Iterations | 17 |
| --- | --- |
| Final SSE | 0.337982 |
| DFE | 20 |
| MSE | 0.0168991 |
| S | 0.129997 |

**Table A2.2.4**: Retrieved records of datamining from Factiva™

| Year | Year-Initial year | Records | Normalized records |
| --- | --- | --- | --- |
| 1991 | 0 | 3 | 0.002762 |
| 1992 | 1 | 1 | 0.000921 |
| 1993 | 2 | 2 | 0.001842 |
| 1994 | 3 | 12 | 0.01105 |
| 1995 | 4 | 27 | 0.024862 |
| 1996 | 5 | 141 | 0.129834 |
| 1997 | 6 | 177 | 0.162983 |
| 1998 | 7 | 131 | 0.120626 |
| 1999 | 8 | 159 | 0.146409 |
| 2000 | 9 | 123 | 0.11326 |
| 2001 | 10 | 122 | 0.112339 |
| 2002 | 11 | 89 | 0.081952 |
| 2003 | 12 | 98 | 0.090239 |
| 2004 | 13 | 92 | 0.084715 |
| 2005 | 14 | 17 | 0.015654 |
| 2006 | 15 | 18 | 0.016575 |
| 2007 | 16 | 54 | 0.049724 |
| 2008 | 17 | 106 | 0.097606 |
| 2009 | 18 | 99 | 0.09116 |
| 2010 | 19 | 145 | 0.133517 |
| 2011 | 20 | 243 | 0.223757 |
| 2012 | 21 | 278 | 0.255985 |
| 2013 | 22 | 392 | 0.360958 |
| 2014 | 23 | 619 | 0.569982 |
| 2015 | 24 | 911 | 0.838858 |
| 2016 | 25 | 1086 | 1 |

**Minitab 18™ summary for hype-type evolution fit of datamining records from *Factiva™***

**Method**

| Algorithm | Marquardt |
| --- | --- |
| Max iterations | 200 |
| Tolerance | 0.00001 |

**Equation**

Datamining from *Factiva™* = 49.1291 * 0.485003 * 1 * EXP(0.485003 * 'Year-Initial_Year') / ((49.1291 + EXP(0.485003 * 'Year-Initial_Year')) ^ 2) + 1.85318 * 1 / (1 + 49.1291 * EXP(-0.485003 * (Year - 16.572 - 1991)))

**Summary**

| Iterations | 14 |
| --- | --- |
| Final SSE | 0.0238680 |
| DFE | 22 |
| MSE | 0.0010849 |
| S | 0.0329379 |

**A2.3 Location aware intelligence**

**Table A2.3.1**: Retrieved records of location aware intelligence from Science Citation Index™

| Year | Year-Initial year | Records | Normalized records |
| --- | --- | --- | --- |
| 1996 | 0 | 1 | 0.032258065 |
| 1998 | 2 | 1 | 0.032258065 |
| 1999 | 3 | 3 | 0.096774194 |
| 2000 | 4 | 3 | 0.096774194 |
| 2001 | 5 | 4 | 0.129032258 |
| 2002 | 6 | 5 | 0.161290323 |
| 2003 | 7 | 13 | 0.419354839 |
| 2004 | 8 | 23 | 0.741935484 |
| 2005 | 9 | 29 | 0.935483871 |
| 2006 | 10 | 23 | 0.741935484 |
| 2007 | 11 | 13 | 0.419354839 |
| 2008 | 12 | 13 | 0.419354839 |
| 2009 | 13 | 13 | 0.419354839 |
| 2010 | 14 | 18 | 0.580645161 |
| 2011 | 15 | 17 | 0.548387097 |
| 2012 | 16 | 19 | 0.612903226 |
| 2013 | 17 | 16 | 0.516129032 |
| 2014 | 18 | 26 | 0.838709677 |
| 2015 | 19 | 28 | 0.903225806 |
| 2016 | 20 | 31 | 1 |

**Minitab 18™ summary for logistic growth fit of location aware intelligence records from *Science Citation Index™***

**Method**

| Algorithm | Marquardt |
| --- | --- |
| Max iterations | 200 |
| Tolerance | 0.00001 |

**Equation**

Location aware intelligence records from SCI = 1 / (1 + 6.69377 * EXP(-0.177617 * 'Year-Initial_Year'))

**Summary**

| Iterations | 14 |
| --- | --- |
| Final SSE | 0.756450 |
| DFE | 18 |
| MSE | 0.0420250 |
| S | 0.205000 |

**Table A2.3.2**: Retrieved records of location aware intelligence from INSPEC™

| Year | Year-Initial year | Records | Normalized records |
| --- | --- | --- | --- |
| 1995 | 0 | 1 | 0.011494253 |
| 1996 | 1 | 1 | 0.011494253 |
| 1997 | 2 | 2 | 0.022988506 |
| 1998 | 3 | 2 | 0.022988506 |
| 1999 | 4 | 5 | 0.057471264 |
| 2000 | 5 | 10 | 0.114942529 |
| 2001 | 6 | 15 | 0.172413793 |
| 2002 | 7 | 18 | 0.206896552 |
| 2003 | 8 | 24 | 0.275862069 |
| 2004 | 9 | 47 | 0.540229885 |
| 2005 | 10 | 61 | 0.701149425 |
| 2006 | 11 | 57 | 0.655172414 |
| 2007 | 12 | 52 | 0.597701149 |
| 2008 | 13 | 79 | 0.908045977 |
| 2009 | 14 | 77 | 0.885057471 |
| 2010 | 15 | 78 | 0.896551724 |
| 2011 | 16 | 64 | 0.735632184 |
| 2012 | 17 | 68 | 0.781609195 |
| 2013 | 18 | 59 | 0.67816092 |
| 2014 | 19 | 60 | 0.689655172 |
| 2015 | 20 | 63 | 0.724137931 |
| 2016 | 21 | 87 | 1 |

**Minitab 18™ summary for logistic growth fit of location aware intelligence records from *INSPEC™***

**Method**

| Algorithm | Marquardt |
| --- | --- |
| Max iterations | 200 |
| Tolerance | 0.00001 |

**Equation**

Location aware intelligence records from *INSPEC™* = 1 / (1 + 23.7158 * EXP(-0.320342 * 'Year-Initial_Year'))

**Summary**

| Iterations | 23 |
| --- | --- |
| Final SSE | 0.360788 |
| DFE | 20 |
| MSE | 0.0180394 |
| S | 0.134311 |

**Table A2.3.3**: Retrieved records of location aware intelligence from Patseer™

| Year | Year-Initial year | Records | Normalized records |
| --- | --- | --- | --- |
| 1996 | 0 | 2 | 0.027777778 |
| 2000 | 4 | 10 | 0.138888889 |
| 2001 | 5 | 12 | 0.166666667 |
| 2002 | 6 | 9 | 0.125 |
| 2003 | 7 | 16 | 0.222222222 |
| 2004 | 8 | 21 | 0.291666667 |
| 2005 | 9 | 35 | 0.486111111 |
| 2006 | 10 | 38 | 0.527777778 |
| 2007 | 11 | 36 | 0.5 |
| 2008 | 12 | 34 | 0.472222222 |
| 2009 | 13 | 45 | 0.625 |
| 2010 | 14 | 47 | 0.652777778 |
| 2011 | 15 | 67 | 0.930555556 |
| 2012 | 16 | 72 | 1 |
| 2013 | 17 | 68 | 0.944444444 |
| 2014 | 18 | 56 | 0.777777778 |
| 2015 | 19 | 48 | 0.666666667 |
| 2016 | 20 | 25 | 0.347222222 |

**Minitab 18™ summary for logistic growth fit of location aware intelligence records from *Patseer™***

**Method**

| Algorithm | Marquardt |
| --- | --- |
| Max iterations | 200 |
| Tolerance | 0.00001 |

**Equation**

Location aware intelligence records from *Patseer™* = 1 / (1 + 12.6071 * EXP(-0.229575 * 'Year-Initial_Year'))

**Summary**

| Iterations | 18 |
| --- | --- |
| Final SSE | 0.507129 |
| DFE | 16 |
| MSE | 0.0316956 |
| S | 0.178033 |

**Table A2.3.4**: Retrieved records of location aware intelligence from Factiva™

| Year | Year-Initial year | Records | Normalized records |
| --- | --- | --- | --- |
| 1998 | 0 | 3 | 0.037037 |
| 2000 | 2 | 12 | 0.148148 |
| 2001 | 3 | 11 | 0.135802 |
| 2002 | 4 | 19 | 0.234568 |
| 2003 | 5 | 10 | 0.123457 |
| 2004 | 6 | 7 | 0.08642 |
| 2005 | 7 | 7 | 0.08642 |
| 2006 | 8 | 7 | 0.08642 |
| 2007 | 9 | 3 | 0.037037 |
| 2008 | 10 | 6 | 0.074074 |
| 2009 | 11 | 7 | 0.08642 |
| 2010 | 12 | 19 | 0.234568 |
| 2011 | 13 | 17 | 0.209877 |
| 2012 | 14 | 16 | 0.197531 |
| 2013 | 15 | 13 | 0.160494 |
| 2014 | 16 | 56 | 0.691358 |
| 2015 | 17 | 73 | 0.901235 |
| 2016 | 18 | 81 | 1 |

**Minitab 18™ summary for hype-type evolution fit of location aware intelligence records from *Factiva™***

**Method**

| Algorithm | Marquardt |
| --- | --- |
| Max iterations | 200 |
| Tolerance | 0.0001 |

**Equation**

Location aware from *Factiva™* = 11.0051 * 0.608837 * 1 * EXP(0.608837 * 'Year-Initial_Year') / ((11.0051 + EXP(0.608837 * 'Year-Initial_Year')) ^ 2) + 1.56769 * 1 / (1 + 11.0051 * EXP(-0.608837 * (Year - 12.8993 - 1998)))

**Summary**

| Iterations | 24 |
| --- | --- |
| Final SSE | 0.112734 |
| DFE | 14 |
| MSE | 0.0080524 |
| S | 0.0897353 |

**A2.4 Microelectromechanical systems**

**Table A2.4.1**: Retrieved records of microelectromechancial systems from Science Citation Index™

| Year | Year-Initial year | Records | Normalized records |
| --- | --- | --- | --- |
| 1963 | 0 | 2 | 0.003257329 |
| 1968 | 5 | 1 | 0.001628664 |
| 1969 | 6 | 1 | 0.001628664 |
| 1970 | 7 | 1 | 0.001628664 |
| 1971 | 8 | 1 | 0.001628664 |
| 1981 | 18 | 1 | 0.001628664 |
| 1983 | 20 | 1 | 0.001628664 |
| 1984 | 21 | 1 | 0.001628664 |
| 1985 | 22 | 2 | 0.003257329 |
| 1988 | 25 | 4 | 0.006514658 |
| 1990 | 27 | 1 | 0.001628664 |
| 1991 | 28 | 3 | 0.004885993 |
| 1992 | 29 | 4 | 0.006514658 |
| 1993 | 30 | 3 | 0.004885993 |
| 1994 | 31 | 8 | 0.013029316 |
| 1995 | 32 | 22 | 0.035830619 |
| 1996 | 33 | 23 | 0.037459283 |
| 1997 | 34 | 53 | 0.086319218 |
| 1998 | 35 | 108 | 0.175895765 |
| 1999 | 36 | 104 | 0.169381107 |
| 2000 | 37 | 146 | 0.237785016 |
| 2001 | 38 | 208 | 0.338762215 |
| 2002 | 39 | 254 | 0.413680782 |
| 2003 | 40 | 341 | 0.555374593 |
| 2004 | 41 | 429 | 0.698697068 |
| 2005 | 42 | 460 | 0.749185668 |
| 2006 | 43 | 474 | 0.771986971 |
| 2007 | 44 | 564 | 0.918566775 |
| 2008 | 45 | 522 | 0.850162866 |
| 2009 | 46 | 538 | 0.876221498 |
| 2010 | 47 | 526 | 0.856677524 |
| 2011 | 48 | 553 | 0.900651466 |
| 2012 | 49 | 602 | 0.980456026 |
| 2013 | 50 | 576 | 0.938110749 |
| 2014 | 51 | 494 | 0.804560261 |
| 2015 | 52 | 614 | 1 |
| 2016 | 53 | 575 | 0.936482085 |

**Minitab 18™ summary for logistic growth fit of microelectromechanical systems records from *Science Citation Index™***

**Method**

| Algorithm | Marquardt |
| --- | --- |
| Max iterations | 200 |
| Tolerance | 0.00001 |

**Equation**

MEMS records from SCI = 1 / (1 + 4.89745e+006 * EXP(-0.38864 * 'Year-Initial_Year'))

**Summary**

| Iterations | 36 |
| --- | --- |
| Final SSE | 0.0722510 |
| DFE | 35 |
| MSE | 0.0020643 |
| S | 0.0454347 |

**Table A2.4.2**: Retrieved records of microelectromechanical systems from INSPEC™

| Years | Year-Initial year | Records | Normalized records |
| --- | --- | --- | --- |
| 1969 | 0 | 1 | 0.000641849 |
| 1970 | 1 | 1 | 0.000641849 |
| 1971 | 2 | 2 | 0.001283697 |
| 1972 | 3 | 1 | 0.000641849 |
| 1973 | 4 | 1 | 0.000641849 |
| 1978 | 9 | 1 | 0.000641849 |
| 1980 | 11 | 1 | 0.000641849 |
| 1981 | 12 | 1 | 0.000641849 |
| 1982 | 13 | 6 | 0.003851091 |
| 1983 | 14 | 7 | 0.00449294 |
| 1985 | 16 | 3 | 0.001925546 |
| 1986 | 17 | 3 | 0.001925546 |
| 1987 | 18 | 1 | 0.000641849 |
| 1988 | 19 | 3 | 0.001925546 |
| 1989 | 20 | 1 | 0.000641849 |
| 1990 | 21 | 5 | 0.003209243 |
| 1991 | 22 | 10 | 0.006418485 |
| 1992 | 23 | 6 | 0.003851091 |
| 1993 | 24 | 7 | 0.00449294 |
| 1994 | 25 | 20 | 0.01283697 |
| 1995 | 26 | 81 | 0.05198973 |
| 1996 | 27 | 109 | 0.069961489 |
| 1997 | 28 | 182 | 0.116816431 |
| 1998 | 29 | 295 | 0.189345315 |
| 1999 | 30 | 364 | 0.233632863 |
| 2000 | 31 | 479 | 0.307445443 |
| 2001 | 32 | 697 | 0.447368421 |
| 2002 | 33 | 781 | 0.501283697 |
| 2003 | 34 | 1075 | 0.689987163 |
| 2004 | 35 | 977 | 0.627086008 |
| 2005 | 36 | 1220 | 0.783055199 |
| 2006 | 37 | 1317 | 0.845314506 |
| 2007 | 38 | 1483 | 0.951861361 |
| 2008 | 39 | 1339 | 0.859435173 |
| 2009 | 40 | 1486 | 0.953786906 |
| 2010 | 41 | 1442 | 0.925545571 |
| 2011 | 42 | 1558 | 1 |
| 2012 | 43 | 1416 | 0.90885751 |
| 2013 | 44 | 1510 | 0.969191271 |
| 2014 | 45 | 1325 | 0.850449294 |
| 2015 | 46 | 1395 | 0.895378691 |
| 2016 | 47 | 1307 | 0.838896021 |

**Minitab 18™ summary for logistic growth fit of microelectromechanical systems records from *INSPEC™***

**Method**

| Algorithm | Marquardt |
| --- | --- |
| Max iterations | 200 |
| Tolerance | 0.00001 |

**Equation**

MEMS records from *INSPEC™* = 1 / (1 + 437974 * EXP(-0.395255 * 'Year-Initial_Year'))

**Summary**

| Iterations | 36 |
| --- | --- |
| Final SSE | 0.0863688 |
| DFE | 40 |
| MSE | 0.0021592 |
| S | 0.0464674 |

**Table A2.4.3**: Retrieved records of microelectromechanical systems from Patseer™

| Year | Year-Initial year | Records | Normalized records |
| --- | --- | --- | --- |
| 1982 | 0 | 4 | 0.021505376 |
| 1992 | 10 | 1 | 0.005376344 |
| 1993 | 11 | 2 | 0.010752688 |
| 1994 | 12 | 2 | 0.010752688 |
| 1995 | 13 | 3 | 0.016129032 |
| 1996 | 14 | 5 | 0.02688172 |
| 1997 | 15 | 9 | 0.048387097 |
| 1998 | 16 | 16 | 0.086021505 |
| 1999 | 17 | 16 | 0.086021505 |
| 2000 | 18 | 39 | 0.209677419 |
| 2001 | 19 | 66 | 0.35483871 |
| 2002 | 20 | 92 | 0.494623656 |
| 2003 | 21 | 91 | 0.489247312 |
| 2004 | 22 | 83 | 0.446236559 |
| 2005 | 23 | 89 | 0.478494624 |
| 2006 | 24 | 77 | 0.413978495 |
| 2007 | 25 | 121 | 0.650537634 |
| 2008 | 26 | 125 | 0.672043011 |
| 2009 | 27 | 112 | 0.602150538 |
| 2010 | 28 | 125 | 0.672043011 |
| 2011 | 29 | 166 | 0.892473118 |
| 2012 | 30 | 154 | 0.827956989 |
| 2013 | 31 | 182 | 0.978494624 |
| 2014 | 32 | 186 | 1 |
| 2015 | 33 | 151 | 0.811827957 |
| 2016 | 34 | 90 | 0.483870968 |

**Minitab 18™ summary for logistic growth fit of microelectromechanical systems records from *Patseer™***

**Method**

| Algorithm | Marquardt |
| --- | --- |
| Max iterations | 200 |
| Tolerance | 0.00001 |

**Equation**

MEMS records from *Patseer™* = 1 / (1 + 213.046 * EXP(-0.229241 * 'Year-Initial_Year'))

**Summary**

| Iterations | 25 |
| --- | --- |
| Final SSE | 0.367983 |
| DFE | 24 |
| MSE | 0.0153326 |
| S | 0.123825 |

**Table A2.4.4**: Retrieved records of microelectromechanical systems from Factiva™

| Year | Year-Initial year | Records | Normalized records |
| --- | --- | --- | --- |
| 1985 | 0 | 1 | 0.001422 |
| 1989 | 4 | 1 | 0.001422 |
| 1991 | 6 | 1 | 0.001422 |
| 1994 | 9 | 11 | 0.015647 |
| 1995 | 10 | 9 | 0.012802 |
| 1996 | 11 | 25 | 0.035562 |
| 1997 | 12 | 42 | 0.059744 |
| 1998 | 13 | 55 | 0.078236 |
| 1999 | 14 | 76 | 0.108108 |
| 2000 | 15 | 137 | 0.194879 |
| 2001 | 16 | 248 | 0.352774 |
| 2002 | 17 | 203 | 0.288762 |
| 2003 | 18 | 215 | 0.305832 |
| 2004 | 19 | 181 | 0.257468 |
| 2005 | 20 | 65 | 0.092461 |
| 2006 | 21 | 80 | 0.113798 |
| 2007 | 22 | 69 | 0.098151 |
| 2008 | 23 | 65 | 0.092461 |
| 2009 | 24 | 115 | 0.163585 |
| 2010 | 25 | 240 | 0.341394 |
| 2011 | 26 | 225 | 0.320057 |
| 2012 | 27 | 244 | 0.347084 |
| 2013 | 28 | 189 | 0.268848 |
| 2014 | 29 | 519 | 0.738265 |
| 2015 | 30 | 703 | 1 |
| 2016 | 31 | 646 | 0.918919 |

**Minitab 18™ summary for hype-type evolution fit of microelectromechanical systems records from *Factiva™***

**Method**

| Algorithm | Marquardt |
| --- | --- |
| Max iterations | 200 |
| Tolerance | 0.0001 |

**Equation**

MEMS from *Factiva™* = 18733.2 * 0.575252 * 1 * EXP(0.575252 * 'Year-Initial_Year') / ((18733.2 + EXP(0.575252 * 'Year-Initial_Year')) ^ 2) + 1.19288 * 1 / (1 + 18733.2 * EXP(-0.575252 * (Year - 11.2808 - 1985)))

**Summary**

| Iterations | 51 |
| --- | --- |
| Final SSE | 0.275237 |
| DFE | 22 |
| MSE | 0.0125108 |
| S | 0.111852 |

**A2.5 Organic light emitting diode**

**Table A2.5.1**: Retrieved records of organic light emitting diode from Science Citation Index™

| Year | Year-Initial year | Records | Normalized records |
| --- | --- | --- | --- |
| 1994 | 0 | 4 | 0.006134969 |
| 1995 | 1 | 8 | 0.012269939 |
| 1996 | 2 | 21 | 0.032208589 |
| 1997 | 3 | 58 | 0.088957055 |
| 1998 | 4 | 65 | 0.099693252 |
| 1999 | 5 | 90 | 0.13803681 |
| 2000 | 6 | 133 | 0.20398773 |
| 2001 | 7 | 152 | 0.233128834 |
| 2002 | 8 | 181 | 0.277607362 |
| 2003 | 9 | 213 | 0.326687117 |
| 2004 | 10 | 252 | 0.386503067 |
| 2005 | 11 | 334 | 0.512269939 |
| 2006 | 12 | 423 | 0.648773006 |
| 2007 | 13 | 440 | 0.674846626 |
| 2008 | 14 | 516 | 0.791411043 |
| 2009 | 15 | 521 | 0.799079755 |
| 2010 | 16 | 499 | 0.765337423 |
| 2011 | 17 | 564 | 0.865030675 |
| 2012 | 18 | 543 | 0.832822086 |
| 2013 | 19 | 581 | 0.891104294 |
| 2014 | 20 | 647 | 0.992331288 |
| 2015 | 21 | 649 | 0.995398773 |
| 2016 | 22 | 652 | 1 |

**Minitab 18™ summary for logistic growth fit of organic light emitting diode records from *Science Citation Index™***

**Method**

| Algorithm | Marquardt |
| --- | --- |
| Max iterations | 200 |
| Tolerance | 0.00001 |

**Equation**

OLED records from SCI = 1 / (1 + 32.2698 * EXP(-0.319652 * 'Year-Initial_Year'))

**Summary**

| Iterations | 15 |
| --- | --- |
| Final SSE | 0.0295340 |
| DFE | 21 |
| MSE | 0.0014064 |
| S | 0.0375018 |

**Table A2.5.2**: Retrieved records of organic light emitting diode from INSPEC™

| Year | Year-Initial year | Records | Normalized records |
| --- | --- | --- | --- |
| 1994 | 0 | 4 | 0.006980803 |
| 1995 | 1 | 11 | 0.019197208 |
| 1996 | 2 | 31 | 0.054101222 |
| 1997 | 3 | 54 | 0.094240838 |
| 1998 | 4 | 86 | 0.15008726 |
| 1999 | 5 | 99 | 0.172774869 |
| 2000 | 6 | 141 | 0.246073298 |
| 2001 | 7 | 176 | 0.307155323 |
| 2002 | 8 | 200 | 0.34904014 |
| 2003 | 9 | 223 | 0.389179756 |
| 2004 | 10 | 280 | 0.488656195 |
| 2005 | 11 | 382 | 0.666666667 |
| 2006 | 12 | 470 | 0.820244328 |
| 2007 | 13 | 499 | 0.870855148 |
| 2008 | 14 | 565 | 0.986038394 |
| 2009 | 15 | 573 | 1 |
| 2010 | 16 | 518 | 0.904013962 |
| 2011 | 17 | 535 | 0.933682373 |
| 2012 | 18 | 521 | 0.909249564 |
| 2013 | 19 | 494 | 0.862129145 |
| 2014 | 20 | 524 | 0.914485166 |
| 2015 | 21 | 495 | 0.863874346 |
| 2016 | 22 | 448 | 0.781849913 |

**Minitab 18™ summary for logistic growth fit of organic light emitting diode records from *INSPEC™***

**Method**

| Algorithm | Marquardt |
| --- | --- |
| Max iterations | 200 |
| Tolerance | 0.00001 |

**Equation**

OLED records from *INSPEC™* = 1 / (1 + 42.696 * EXP(-0.405221 * 'Year-Initial_Year'))

**Summary**

| Iterations | 17 |
| --- | --- |
| Final SSE | 0.134932 |
| DFE | 21 |
| MSE | 0.0064253 |
| S | 0.0801581 |

**Table A2.5.3**: Retrieved records of organic light emitting diode from Patseer™

| Year | Year-Initial year | Records | Normalized records |
| --- | --- | --- | --- |
| 1990 | 0 | 1 | 0.000422833 |
| 1991 | 1 | 1 | 0.000422833 |
| 1993 | 3 | 1 | 0.000422833 |
| 1994 | 4 | 2 | 0.000845666 |
| 1995 | 5 | 14 | 0.005919662 |
| 1996 | 6 | 21 | 0.008879493 |
| 1997 | 7 | 66 | 0.027906977 |
| 1998 | 8 | 70 | 0.029598309 |
| 1999 | 9 | 60 | 0.025369979 |
| 2000 | 10 | 100 | 0.042283298 |
| 2001 | 11 | 219 | 0.092600423 |
| 2002 | 12 | 332 | 0.14038055 |
| 2003 | 13 | 527 | 0.222832981 |
| 2004 | 14 | 697 | 0.294714588 |
| 2005 | 15 | 930 | 0.393234672 |
| 2006 | 16 | 990 | 0.418604651 |
| 2007 | 17 | 898 | 0.379704017 |
| 2008 | 18 | 827 | 0.349682875 |
| 2009 | 19 | 903 | 0.381818182 |
| 2010 | 20 | 1173 | 0.495983087 |
| 2011 | 21 | 1251 | 0.528964059 |
| 2012 | 22 | 1577 | 0.666807611 |
| 2013 | 23 | 2365 | 1 |
| 2014 | 24 | 2248 | 0.950528541 |
| 2015 | 25 | 2322 | 0.981818182 |
| 2016 | 26 | 1637 | 0.69217759 |

**Minitab 18™ summary for logistic growth fit of organic light emitting diode records from *Patseer™***

**Method**

| Algorithm | Marquardt |
| --- | --- |
| Max iterations | 200 |
| Tolerance | 0.00001 |

**Equation**

OLED records from *Patseer™* = 1 / (1 + 166.359 * EXP(-0.273154 * 'Year-Initial_Year'))

**Summary**

| Iterations | 22 |
| --- | --- |
| Final SSE | 0.223167 |
| DFE | 24 |
| MSE | 0.0092986 |
| S | 0.0964294 |

**Table A2.5.4**: Retrieved records of organic light emitting diode from Factiva™

| Year | Year-Initial year | Records | Normalized records |
| --- | --- | --- | --- |
| 1996 | 0 | 1 | 0.000623 |
| 1998 | 2 | 2 | 0.001247 |
| 1999 | 3 | 6 | 0.003741 |
| 2000 | 4 | 47 | 0.029302 |
| 2001 | 5 | 83 | 0.051746 |
| 2002 | 6 | 91 | 0.056733 |
| 2003 | 7 | 105 | 0.065461 |
| 2004 | 8 | 91 | 0.056733 |
| 2005 | 9 | 66 | 0.041147 |
| 2006 | 10 | 38 | 0.023691 |
| 2007 | 11 | 45 | 0.028055 |
| 2008 | 12 | 91 | 0.056733 |
| 2009 | 13 | 104 | 0.064838 |
| 2010 | 14 | 154 | 0.09601 |
| 2011 | 15 | 168 | 0.104738 |
| 2012 | 16 | 355 | 0.221322 |
| 2013 | 17 | 400 | 0.249377 |
| 2014 | 18 | 839 | 0.523067 |
| 2015 | 19 | 1344 | 0.837905 |
| 2016 | 20 | 1604 | 1 |

**Minitab 18™ summary for hype-type evolution fit of organic light emitting diode records from *Factiva™***

**Method**

| Algorithm | Marquardt |
| --- | --- |
| Max iterations | 200 |
| Tolerance | 0.0001 |

**Equation**

OLED from *Factiva™* = 36.7973 * 0.524603 * 1 * EXP(0.524603 * 'Year-Initial_Year') / ((36.7973 + EXP(0.524603 * 'Year-Initial_Year')) ^ 2) + 2.38021 * 1 / (1 + 36.7973 * EXP(-0.524603 * (Year - 13.6111 - 1996)))

**Summary**

| Iterations | 18 |
| --- | --- |
| Final SSE | 0.0488021 |
| DFE | 16 |
| MSE | 0.0030501 |
| S | 0.0552280 |

**A2.6 RFID**

**Table A2.6.1**: Retrieved records of RFID from Science Citation Index™

| Year | Year-Initial year | Records | Normalized records |
| --- | --- | --- | --- |
| 1984 | 0 | 1 | 0.002304147 |
| 1985 | 1 | 2 | 0.004608295 |
| 1994 | 10 | 2 | 0.004608295 |
| 1995 | 11 | 2 | 0.004608295 |
| 1996 | 12 | 3 | 0.006912442 |
| 1997 | 13 | 6 | 0.013824885 |
| 1998 | 14 | 5 | 0.011520737 |
| 1999 | 15 | 9 | 0.020737327 |
| 2000 | 16 | 5 | 0.011520737 |
| 2001 | 17 | 6 | 0.013824885 |
| 2002 | 18 | 10 | 0.023041475 |
| 2003 | 19 | 24 | 0.055299539 |
| 2004 | 20 | 57 | 0.131336406 |
| 2005 | 21 | 129 | 0.297235023 |
| 2006 | 22 | 230 | 0.529953917 |
| 2007 | 23 | 223 | 0.513824885 |
| 2008 | 24 | 231 | 0.532258065 |
| 2009 | 25 | 325 | 0.748847926 |
| 2010 | 26 | 380 | 0.875576037 |
| 2011 | 27 | 434 | 1 |
| 2012 | 28 | 427 | 0.983870968 |
| 2013 | 29 | 409 | 0.942396313 |
| 2014 | 30 | 421 | 0.970046083 |
| 2015 | 31 | 379 | 0.873271889 |
| 2016 | 32 | 364 | 0.838709677 |

**Minitab 18™ summary for logistic growth fit of RFID records from *Science Citation Index™***

**Method**

| Algorithm | Marquardt |
| --- | --- |
| Max iterations | 200 |
| Tolerance | 0.00001 |

**Equation**

RFID records from SCI = 1 / (1 + 297325 * EXP(-0.552117 * 'Year-Initial_Year'))

**Summary**

| Iterations | 40 |
| --- | --- |
| Final SSE | 0.0927869 |
| DFE | 23 |
| MSE | 0.0040342 |
| S | 0.0635155 |

**Table A2.6.2**: Retrieved records of RFID from INSPEC™

| Year | Year-Initial year | Records | Normalized records |
| --- | --- | --- | --- |
| 1988 | 0 | 2 | 0.001218027 |
| 1990 | 2 | 1 | 0.000609013 |
| 1993 | 5 | 5 | 0.003045067 |
| 1994 | 6 | 5 | 0.003045067 |
| 1995 | 7 | 6 | 0.00365408 |
| 1996 | 8 | 5 | 0.003045067 |
| 1997 | 9 | 6 | 0.00365408 |
| 1998 | 10 | 5 | 0.003045067 |
| 1999 | 11 | 27 | 0.016443362 |
| 2000 | 12 | 15 | 0.009135201 |
| 2001 | 13 | 20 | 0.012180268 |
| 2002 | 14 | 45 | 0.027405603 |
| 2003 | 15 | 54 | 0.032886724 |
| 2004 | 16 | 187 | 0.113885505 |
| 2005 | 17 | 376 | 0.228989038 |
| 2006 | 18 | 679 | 0.413520097 |
| 2007 | 19 | 1104 | 0.672350792 |
| 2008 | 20 | 1303 | 0.793544458 |
| 2009 | 21 | 1602 | 0.975639464 |
| 2010 | 22 | 1642 | 1 |
| 2011 | 23 | 1609 | 0.979902558 |
| 2012 | 24 | 1445 | 0.880024361 |
| 2013 | 25 | 1413 | 0.860535932 |
| 2014 | 26 | 1342 | 0.817295981 |
| 2015 | 27 | 1132 | 0.689403167 |
| 2016 | 28 | 998 | 0.607795371 |

**Minitab 18™ summary for logistic growth fit of RFID records from *INSPEC™***

**Method**

| Algorithm | Marquardt |
| --- | --- |
| Max iterations | 200 |
| Tolerance | 0.00001 |

**Equation**

RFID records from *INSPEC™* = 1 / (1 + 2.30206e+007 * EXP(-0.925658 * 'Year-Initial_Year'))

**Summary**

| Iterations | 61 |
| --- | --- |
| Final SSE | 0.321265 |
| DFE | 24 |
| MSE | 0.0133861 |
| S | 0.115698 |

**Table A2.6.3**: Retrieved records of RFID from Patseer™

| Year | Year-Initial year | Records | Normalized records |
| --- | --- | --- | --- |
| 1980 | 0 | 1 | 0.000462749 |
| 1988 | 8 | 1 | 0.000462749 |
| 1991 | 11 | 1 | 0.000462749 |
| 1992 | 12 | 5 | 0.002313744 |
| 1993 | 13 | 8 | 0.00370199 |
| 1994 | 14 | 11 | 0.005090236 |
| 1995 | 15 | 22 | 0.010180472 |
| 1996 | 16 | 23 | 0.010643221 |
| 1997 | 17 | 42 | 0.019435447 |
| 1998 | 18 | 65 | 0.030078667 |
| 1999 | 19 | 132 | 0.061082832 |
| 2000 | 20 | 128 | 0.059231837 |
| 2001 | 21 | 204 | 0.09440074 |
| 2002 | 22 | 226 | 0.104581212 |
| 2003 | 23 | 415 | 0.192040722 |
| 2004 | 24 | 844 | 0.390559926 |
| 2005 | 25 | 1685 | 0.779731606 |
| 2006 | 26 | 2161 | 1 |
| 2007 | 27 | 2141 | 0.990745025 |
| 2008 | 28 | 1976 | 0.914391485 |
| 2009 | 29 | 1686 | 0.780194354 |
| 2010 | 30 | 1566 | 0.724664507 |
| 2011 | 31 | 1492 | 0.690421101 |
| 2012 | 32 | 1608 | 0.744099954 |
| 2013 | 33 | 1580 | 0.731142989 |
| 2014 | 34 | 1445 | 0.668671911 |
| 2015 | 35 | 1412 | 0.653401203 |
| 2016 | 36 | 1148 | 0.531235539 |

**Minitab 18™ summary for logistic growth fit of RFID records from *Patseer™***

**Method**

| Algorithm | Marquardt |
| --- | --- |
| Max iterations | 200 |
| Tolerance | 0.00001 |

**Equation**

RFID records from *Patseer™* = 1 / (1 + 8.2641e+013 * EXP(-1.32718 * 'Year-Initial_Year'))

**Summary**

| Iterations | 164 |
| --- | --- |
| Final SSE | 0.840432 |
| DFE | 26 |
| MSE | 0.0323243 |
| S | 0.179790 |

**Table A2.6.4**: Retrieved records of RFID from Factiva™

| Year | Year-Initial year | Records | Normalized records |
| --- | --- | --- | --- |
| 1991 | 0 | 2 | 0.001092 |
| 1992 | 1 | 2 | 0.001092 |
| 1993 | 2 | 6 | 0.003277 |
| 1994 | 3 | 4 | 0.002185 |
| 1995 | 4 | 7 | 0.003823 |
| 1996 | 5 | 14 | 0.007646 |
| 1997 | 6 | 30 | 0.016384 |
| 1998 | 7 | 46 | 0.025123 |
| 1999 | 8 | 91 | 0.0497 |
| 2000 | 9 | 129 | 0.070453 |
| 2001 | 10 | 120 | 0.065538 |
| 2002 | 11 | 98 | 0.053523 |
| 2003 | 12 | 569 | 0.310759 |
| 2004 | 13 | 1831 | 1 |
| 2005 | 14 | 1182 | 0.645549 |
| 2006 | 15 | 803 | 0.438558 |
| 2007 | 16 | 545 | 0.297652 |
| 2008 | 17 | 436 | 0.238121 |
| 2009 | 18 | 474 | 0.258875 |
| 2010 | 19 | 525 | 0.286729 |
| 2011 | 20 | 366 | 0.199891 |
| 2012 | 21 | 474 | 0.258875 |
| 2013 | 22 | 334 | 0.182414 |
| 2014 | 23 | 934 | 0.510104 |
| 2015 | 24 | 817 | 0.446204 |
| 2016 | 25 | 735 | 0.40142 |

**Minitab 18™ summary for hype-type evolution fit of RFID records from *Factiva™***

**Method**

| Algorithm | Marquardt |
| --- | --- |
| Max iterations | 200 |
| Tolerance | 0.0001 |

**Equation**

RFID from *Factiva™* = 22798.4 * 1.25816 * 1 * EXP(1.25816 * 'Year-Initial_Year') / ((22798.4 + EXP(1.25816 * 'Year-Initial_Year')) ^ 2) + 0.389286 * 1 / (1 + 22798.4 * EXP(-1.25816 * (Year - 3.21005 - 1991)))

**Summary**

| Iterations | 130 |
| --- | --- |
| Final SSE | 0.837177 |
| DFE | 22 |
| MSE | 0.0380535 |
| S | 0.195073 |

###

**A2.7 Smartphone**

**Table A2.7.1**: Retrieved records of smartphone from Science Citation Index™

| Year | Year-Initial year | Records | Normalized records |
| --- | --- | --- | --- |
| 2002 | 0 | 1 | 0.001318 |
| 2003 | 1 | 1 | 0.001318 |
| 2004 | 2 | 5 | 0.006588 |
| 2005 | 3 | 5 | 0.006588 |
| 2006 | 4 | 7 | 0.009223 |
| 2007 | 5 | 4 | 0.00527 |
| 2008 | 6 | 8 | 0.01054 |
| 2009 | 7 | 7 | 0.009223 |
| 2010 | 8 | 25 | 0.032938 |
| 2011 | 9 | 63 | 0.083004 |
| 2012 | 10 | 151 | 0.198946 |
| 2013 | 11 | 266 | 0.350461 |
| 2014 | 12 | 387 | 0.509881 |
| 2015 | 13 | 569 | 0.749671 |
| 2016 | 14 | 759 | 1 |

**Minitab 18™ summary for logistic growth fit of smartphone records from *Science Citation Index™***

**Method**

| Algorithm | Marquardt |
| --- | --- |
| Max iterations | 200 |
| Tolerance | 0.00001 |

**Equation**

Smartphone records from SCI = 1 / (1 + 65052.3 * EXP(-0.946203 * 'Year-Initial_Year'))

**Summary**

| Iterations | 33 |
| --- | --- |
| Final SSE | 0.0160982 |
| DFE | 13 |
| MSE | 0.0012383 |
| S | 0.0351899 |

**Table A2.7.2**: Retrieved records of smartphone from INSPEC™

| Year | Year-Initial year | Records | Normalized records |
| --- | --- | --- | --- |
| 1999 | 0 | 4 | 0.005277045 |
| 2000 | 1 | 1 | 0.001319261 |
| 2001 | 2 | 1 | 0.001319261 |
| 2002 | 3 | 2 | 0.002638522 |
| 2003 | 4 | 1 | 0.001319261 |
| 2004 | 5 | 9 | 0.011873351 |
| 2005 | 6 | 12 | 0.015831135 |
| 2006 | 7 | 9 | 0.011873351 |
| 2007 | 8 | 14 | 0.018469657 |
| 2008 | 9 | 23 | 0.030343008 |
| 2009 | 10 | 41 | 0.05408971 |
| 2010 | 11 | 87 | 0.114775726 |
| 2011 | 12 | 200 | 0.263852243 |
| 2012 | 13 | 368 | 0.485488127 |
| 2013 | 14 | 513 | 0.676781003 |
| 2014 | 15 | 615 | 0.811345646 |
| 2015 | 16 | 717 | 0.94591029 |
| 2016 | 17 | 758 | 1 |

**Minitab 18™ summary for logistic growth fit of smartphone records from *INSPEC™***

**Method**

| Algorithm | Marquardt |
| --- | --- |
| Max iterations | 200 |
| Tolerance | 0.00001 |

**Equation**

Smartphone records from *INSPEC™* = 1 / (1 + 129568 * EXP(-0.895593 * 'Year-Initial_Year'))

**Summary**

| Iterations | 28 |
| --- | --- |
| Final SSE | 0.0031767 |
| DFE | 16 |
| MSE | 0.0001985 |
| S | 0.0140906 |

**Table A2.7.3**: Retrieved records of smartphone from Patseer™

| Year | Year-Initial year | Records | Normalized records |
| --- | --- | --- | --- |
| 1995 | 0 | 2 | 0.001964637 |
| 1996 | 1 | 1 | 0.000982318 |
| 1997 | 2 | 13 | 0.012770138 |
| 1998 | 3 | 2 | 0.001964637 |
| 1999 | 4 | 4 | 0.003929273 |
| 2000 | 5 | 1 | 0.000982318 |
| 2001 | 6 | 5 | 0.004911591 |
| 2002 | 7 | 6 | 0.00589391 |
| 2003 | 8 | 8 | 0.007858546 |
| 2004 | 9 | 22 | 0.021611002 |
| 2005 | 10 | 24 | 0.023575639 |
| 2006 | 11 | 21 | 0.020628684 |
| 2007 | 12 | 9 | 0.008840864 |
| 2008 | 13 | 12 | 0.011787819 |
| 2009 | 14 | 33 | 0.032416503 |
| 2010 | 15 | 357 | 0.350687623 |
| 2011 | 16 | 626 | 0.614931238 |
| 2012 | 17 | 881 | 0.865422397 |
| 2013 | 18 | 990 | 0.972495088 |
| 2014 | 19 | 1018 | 1 |
| 2015 | 20 | 986 | 0.968565815 |
| 2016 | 21 | 607 | 0.596267191 |

**Minitab 18™ summary for logistic growth fit of smartphone records from *Patseer™***

**Method**

| Algorithm | Marquardt |
| --- | --- |
| Max iterations | 200 |
| Tolerance | 0.00001 |

**Equation**

Smartphone records from *Patseer™* = 1 / (1 + 2.4938e+009 * EXP(-1.38604 * 'Year-Initial_Year'))

**Summary**

| Iterations | 85 |
| --- | --- |
| Final SSE | 0.172449 |
| DFE | 20 |
| MSE | 0.0086224 |
| S | 0.0928571 |

**Table A2.7.4**: Retrieved records of smartphone from Factiva™

| Year | Year-Initial year | Records | Normalized records |
| --- | --- | --- | --- |
| 1984 | 0 | 1 | 0.000112 |
| 1987 | 3 | 1 | 0.000112 |
| 1990 | 6 | 1 | 0.000112 |
| 1991 | 7 | 9 | 0.001008 |
| 1992 | 8 | 4 | 0.000448 |
| 1993 | 9 | 9 | 0.001008 |
| 1994 | 10 | 18 | 0.002016 |
| 1995 | 11 | 19 | 0.002128 |
| 1996 | 12 | 54 | 0.006047 |
| 1997 | 13 | 62 | 0.006943 |
| 1998 | 14 | 51 | 0.005711 |
| 1999 | 15 | 45 | 0.005039 |
| 2000 | 16 | 64 | 0.007167 |
| 2001 | 17 | 92 | 0.010302 |
| 2002 | 18 | 129 | 0.014446 |
| 2003 | 19 | 298 | 0.033371 |
| 2004 | 20 | 280 | 0.031355 |
| 2005 | 21 | 128 | 0.014334 |
| 2006 | 22 | 83 | 0.009295 |
| 2007 | 23 | 99 | 0.011086 |
| 2008 | 24 | 190 | 0.021277 |
| 2009 | 25 | 822 | 0.092049 |
| 2010 | 26 | 2428 | 0.271892 |
| 2011 | 27 | 4982 | 0.557895 |
| 2012 | 28 | 5820 | 0.651736 |
| 2013 | 29 | 8930 | 1 |
| 2014 | 30 | 6660 | 0.745801 |
| 2015 | 31 | 2022 | 0.226428 |
| 2016 | 32 | 1993 | 0.22318 |

**Minitab 18™ summary for hype-type evolution fit of smartphone records from *Factiva™***

**Method**

| Algorithm | Marquardt |
| --- | --- |
| Max iterations | 200 |
| Tolerance | 0.0001 |

**Equation**

Smartphone from *Factiva™* = 3.69852e+006 * 3.36072 * 1 * EXP(3.36072 * 'Year-Initial_Year') / ((3.69852e+006 + EXP(3.36072 * 'Year-Initial_Year')) ^ 2) + 0.570319 * 1 / (1 + 3.69852e+006 * EXP(-3.36072 * (Year - 21.505 - 1984)))

**Summary**

| Iterations | 37 |
| --- | --- |
| Final SSE | 0.470845 |
| DFE | 25 |
| MSE | 0.0188338 |
| S | 0.137236 |

**A2.8 Speech recognition**

**Table A2.8.1**: Retrieved records of speech recognition from Science Citation Index™

| Year | Year-Initial year | Records | Normalized records |
| --- | --- | --- | --- |
| 1959 | 0 | 1 | 0.007463 |
| 1960 | 1 | 5 | 0.037313 |
| 1962 | 3 | 3 | 0.022388 |
| 1963 | 4 | 1 | 0.007463 |
| 1964 | 5 | 5 | 0.037313 |
| 1965 | 6 | 3 | 0.022388 |
| 1966 | 7 | 3 | 0.022388 |
| 1967 | 8 | 5 | 0.037313 |
| 1968 | 9 | 5 | 0.037313 |
| 1969 | 10 | 7 | 0.052239 |
| 1970 | 11 | 12 | 0.089552 |
| 1971 | 12 | 8 | 0.059701 |
| 1972 | 13 | 6 | 0.044776 |
| 1973 | 14 | 6 | 0.044776 |
| 1974 | 15 | 9 | 0.067164 |
| 1975 | 16 | 10 | 0.074627 |
| 1976 | 17 | 13 | 0.097015 |
| 1977 | 18 | 9 | 0.067164 |
| 1978 | 19 | 14 | 0.104478 |
| 1979 | 20 | 13 | 0.097015 |
| 1980 | 21 | 9 | 0.067164 |
| 1981 | 22 | 6 | 0.044776 |
| 1982 | 23 | 16 | 0.119403 |
| 1983 | 24 | 30 | 0.223881 |
| 1984 | 25 | 25 | 0.186567 |
| 1985 | 26 | 27 | 0.201493 |
| 1986 | 27 | 21 | 0.156716 |
| 1987 | 28 | 23 | 0.171642 |
| 1988 | 29 | 18 | 0.134328 |
| 1989 | 30 | 22 | 0.164179 |
| 1990 | 31 | 31 | 0.231343 |
| 1991 | 32 | 47 | 0.350746 |
| 1992 | 33 | 29 | 0.216418 |
| 1993 | 34 | 41 | 0.30597 |
| 1994 | 35 | 77 | 0.574627 |
| 1995 | 36 | 56 | 0.41791 |
| 1996 | 37 | 72 | 0.537313 |
| 1997 | 38 | 63 | 0.470149 |
| 1998 | 39 | 72 | 0.537313 |
| 1999 | 40 | 82 | 0.61194 |
| 2000 | 41 | 103 | 0.768657 |
| 2001 | 42 | 78 | 0.58209 |
| 2002 | 43 | 93 | 0.69403 |
| 2003 | 44 | 104 | 0.776119 |
| 2004 | 45 | 108 | 0.80597 |
| 2005 | 46 | 121 | 0.902985 |
| 2006 | 47 | 134 | 1 |
| 2007 | 48 | 114 | 0.850746 |
| 2008 | 49 | 88 | 0.656716 |
| 2009 | 50 | 93 | 0.69403 |
| 2010 | 51 | 116 | 0.865672 |
| 2011 | 52 | 118 | 0.880597 |
| 2012 | 53 | 113 | 0.843284 |
| 2013 | 54 | 127 | 0.947761 |
| 2014 | 55 | 113 | 0.843284 |
| 2015 | 56 | 99 | 0.738806 |
| 2016 | 57 | 119 | 0.88806 |

**Minitab 18™ summary for logistic growth fit of smartphone records from *Science Citation Index™***

**Method**

| Algorithm | Marquardt |
| --- | --- |
| Max iterations | 200 |
| Tolerance | 0.00001 |

**Equation**

Speech recognition records from SCI = 1 / (1 + 184.639 * EXP(-0.139342 * 'Year-Initial_Year'))

**Summary**

| Iterations | 24 |
| --- | --- |
| Final SSE | 0.315386 |
| DFE | 55 |
| MSE | 0.0057343 |
| S | 0.0757251 |

**Table A2.8.2**: Retrieved records of speech recognition from INSPEC™

| Year | Year-Initial year | Records | Normalized records |
| --- | --- | --- | --- |
| 1969 | 0 | 11 | 0.026004728 |
| 1970 | 1 | 22 | 0.052009456 |
| 1971 | 2 | 14 | 0.033096927 |
| 1972 | 3 | 15 | 0.035460993 |
| 1973 | 4 | 18 | 0.042553191 |
| 1974 | 5 | 13 | 0.030732861 |
| 1975 | 6 | 16 | 0.037825059 |
| 1976 | 7 | 24 | 0.056737589 |
| 1977 | 8 | 18 | 0.042553191 |
| 1978 | 9 | 43 | 0.101654846 |
| 1979 | 10 | 26 | 0.061465721 |
| 1980 | 11 | 40 | 0.094562648 |
| 1981 | 12 | 45 | 0.106382979 |
| 1982 | 13 | 56 | 0.132387707 |
| 1983 | 14 | 93 | 0.219858156 |
| 1984 | 15 | 91 | 0.215130024 |
| 1985 | 16 | 124 | 0.293144208 |
| 1986 | 17 | 139 | 0.328605201 |
| 1987 | 18 | 135 | 0.319148936 |
| 1988 | 19 | 179 | 0.423167849 |
| 1989 | 20 | 232 | 0.548463357 |
| 1990 | 21 | 192 | 0.453900709 |
| 1991 | 22 | 273 | 0.645390071 |
| 1992 | 23 | 225 | 0.531914894 |
| 1993 | 24 | 170 | 0.401891253 |
| 1994 | 25 | 334 | 0.789598109 |
| 1995 | 26 | 196 | 0.463356974 |
| 1996 | 27 | 344 | 0.813238771 |
| 1997 | 28 | 231 | 0.546099291 |
| 1998 | 29 | 289 | 0.68321513 |
| 1999 | 30 | 225 | 0.531914894 |
| 2000 | 31 | 264 | 0.624113475 |
| 2001 | 32 | 242 | 0.572104019 |
| 2002 | 33 | 296 | 0.699763593 |
| 2003 | 34 | 287 | 0.678486998 |
| 2004 | 35 | 359 | 0.848699764 |
| 2005 | 36 | 304 | 0.718676123 |
| 2006 | 37 | 369 | 0.872340426 |
| 2007 | 38 | 351 | 0.829787234 |
| 2008 | 39 | 402 | 0.95035461 |
| 2009 | 40 | 397 | 0.938534279 |
| 2010 | 41 | 423 | 1 |
| 2011 | 42 | 383 | 0.905437352 |
| 2012 | 43 | 386 | 0.912529551 |
| 2013 | 44 | 361 | 0.853427896 |
| 2014 | 45 | 393 | 0.929078014 |
| 2015 | 46 | 330 | 0.780141844 |
| 2016 | 47 | 396 | 0.936170213 |

**Minitab 18™ summary for logistic growth fit of smartphone records from *INSPEC™***

**Method**

| Algorithm | Marquardt |
| --- | --- |
| Max iterations | 200 |
| Tolerance | 0.00001 |

**Equation**

Speech recognition records from *INSPEC™* = 1 / (1 + 20.0337 * EXP(-0.125624 * 'Year-Initial_Year'))

**Summary**

| Iterations | 16 |
| --- | --- |
| Final SSE | 0.402781 |
| DFE | 46 |
| MSE | 0.0087561 |
| S | 0.0935740 |

**Table A2.8.3**: Retrieved records of speech recognition from Patseer™

| Year | Year-Initial year | Records | Normalized records |
| --- | --- | --- | --- |
| 1961 | 0 | 1 | 0.002392344 |
| 1963 | 2 | 1 | 0.002392344 |
| 1964 | 3 | 2 | 0.004784689 |
| 1965 | 4 | 7 | 0.016746411 |
| 1966 | 5 | 8 | 0.019138756 |
| 1967 | 6 | 5 | 0.011961722 |
| 1968 | 7 | 3 | 0.007177033 |
| 1969 | 8 | 3 | 0.007177033 |
| 1970 | 9 | 5 | 0.011961722 |
| 1971 | 10 | 4 | 0.009569378 |
| 1972 | 11 | 3 | 0.007177033 |
| 1973 | 12 | 3 | 0.007177033 |
| 1974 | 13 | 5 | 0.011961722 |
| 1975 | 14 | 4 | 0.009569378 |
| 1976 | 15 | 7 | 0.016746411 |
| 1977 | 16 | 5 | 0.011961722 |
| 1978 | 17 | 14 | 0.033492823 |
| 1979 | 18 | 5 | 0.011961722 |
| 1980 | 19 | 11 | 0.026315789 |
| 1981 | 20 | 20 | 0.04784689 |
| 1982 | 21 | 40 | 0.09569378 |
| 1983 | 22 | 37 | 0.088516746 |
| 1984 | 23 | 36 | 0.086124402 |
| 1985 | 24 | 45 | 0.107655502 |
| 1986 | 25 | 53 | 0.126794258 |
| 1987 | 26 | 78 | 0.186602871 |
| 1988 | 27 | 84 | 0.200956938 |
| 1989 | 28 | 75 | 0.179425837 |
| 1990 | 29 | 88 | 0.210526316 |
| 1991 | 30 | 100 | 0.23923445 |
| 1992 | 31 | 133 | 0.318181818 |
| 1993 | 32 | 124 | 0.296650718 |
| 1994 | 33 | 143 | 0.342105263 |
| 1995 | 34 | 164 | 0.392344498 |
| 1996 | 35 | 206 | 0.492822967 |
| 1997 | 36 | 260 | 0.622009569 |
| 1998 | 37 | 308 | 0.736842105 |
| 1999 | 38 | 345 | 0.825358852 |
| 2000 | 39 | 347 | 0.830143541 |
| 2001 | 40 | 396 | 0.947368421 |
| 2002 | 41 | 355 | 0.849282297 |
| 2003 | 42 | 418 | 1 |
| 2004 | 43 | 390 | 0.933014354 |
| 2005 | 44 | 343 | 0.820574163 |
| 2006 | 45 | 295 | 0.705741627 |
| 2007 | 46 | 309 | 0.73923445 |
| 2008 | 47 | 318 | 0.76076555 |
| 2009 | 48 | 252 | 0.602870813 |
| 2010 | 49 | 217 | 0.519138756 |
| 2011 | 50 | 199 | 0.476076555 |
| 2012 | 51 | 330 | 0.789473684 |
| 2013 | 52 | 345 | 0.825358852 |
| 2014 | 53 | 302 | 0.722488038 |
| 2015 | 54 | 364 | 0.870813397 |
| 2016 | 55 | 292 | 0.698564593 |

**Minitab 18™ summary for logistic growth fit of smartphone records from *Patseer™***

**Method**

| Algorithm | Marquardt |
| --- | --- |
| Max iterations | 200 |
| Tolerance | 0.00001 |

**Equation**

Speech recognition records from *Patseer™* = 1 / (1 + 187.598 * EXP(-0.148403 * 'Year-Initial_Year'))

**Summary**

| Iterations | 30 |
| --- | --- |
| Final SSE | 0.937589 |
| DFE | 53 |
| MSE | 0.0176904 |
| S | 0.133005 |

**Table A2.8.4**: Retrieved records of speech recognition from Factiva™

| Year | Year-Initial year | Records | Normalized records |
| --- | --- | --- | --- |
| 1979 | 0 | 1 | 0.003175 |
| 1981 | 2 | 1 | 0.003175 |
| 1982 | 3 | 3 | 0.009524 |
| 1983 | 4 | 7 | 0.022222 |
| 1984 | 5 | 5 | 0.015873 |
| 1985 | 6 | 2 | 0.006349 |
| 1986 | 7 | 11 | 0.034921 |
| 1987 | 8 | 11 | 0.034921 |
| 1988 | 9 | 11 | 0.034921 |
| 1989 | 10 | 10 | 0.031746 |
| 1990 | 11 | 10 | 0.031746 |
| 1991 | 12 | 11 | 0.034921 |
| 1992 | 13 | 34 | 0.107937 |
| 1993 | 14 | 77 | 0.244444 |
| 1994 | 15 | 126 | 0.4 |
| 1995 | 16 | 166 | 0.526984 |
| 1996 | 17 | 135 | 0.428571 |
| 1997 | 18 | 153 | 0.485714 |
| 1998 | 19 | 282 | 0.895238 |
| 1999 | 20 | 315 | 1 |
| 2000 | 21 | 239 | 0.75873 |
| 2001 | 22 | 246 | 0.780952 |
| 2002 | 23 | 163 | 0.51746 |
| 2003 | 24 | 154 | 0.488889 |
| 2004 | 25 | 92 | 0.292063 |
| 2005 | 26 | 34 | 0.107937 |
| 2006 | 27 | 27 | 0.085714 |
| 2007 | 28 | 21 | 0.066667 |
| 2008 | 29 | 15 | 0.047619 |
| 2009 | 30 | 33 | 0.104762 |
| 2010 | 31 | 70 | 0.222222 |
| 2011 | 32 | 56 | 0.177778 |
| 2012 | 33 | 65 | 0.206349 |
| 2013 | 34 | 28 | 0.088889 |
| 2014 | 35 | 174 | 0.552381 |
| 2015 | 36 | 244 | 0.774603 |
| 2016 | 37 | 296 | 0.939683 |

**Minitab 18™ summary for hype-type evolution fit of smartphone records from *Factiva™***

**Method**

| Algorithm | Marquardt |
| --- | --- |
| Max iterations | 200 |
| Tolerance | 0.0001 |

**Equation**

Speech recognition from *Factiva™* = 654.974 * 0.876075 * 1 * EXP(0.876075 * 'Year-Initial_Year') / ((654.974 + EXP(0.876075 * 'Year-Initial_Year')) ^ 2) + 0.436954 * 1 / (1 + 654.974 * EXP(-0.876075 * (Year - 6.24975 - 1979)))

**Summary**

| Iterations | 126 |
| --- | --- |
| Final SSE | 2.22444 |
| DFE | 33 |
| MSE | 0.0674073 |
| S | 0.259629 |

**A2.9 Text to speech**

**Table A2.9.1**: Retrieved records of text to speech from Science Citation Index™

| Year | Year-Initial year | Records | Normalized records |
| --- | --- | --- | --- |
| 1957 | 0 | 1 | 0.03125 |
| 1958 | 1 | 2 | 0.0625 |
| 1960 | 3 | 3 | 0.09375 |
| 1961 | 4 | 2 | 0.0625 |
| 1962 | 5 | 1 | 0.03125 |
| 1963 | 6 | 1 | 0.03125 |
| 1964 | 7 | 2 | 0.0625 |
| 1965 | 8 | 1 | 0.03125 |
| 1966 | 9 | 3 | 0.09375 |
| 1967 | 10 | 4 | 0.125 |
| 1968 | 11 | 4 | 0.125 |
| 1969 | 12 | 5 | 0.15625 |
| 1970 | 13 | 2 | 0.0625 |
| 1971 | 14 | 5 | 0.15625 |
| 1972 | 15 | 4 | 0.125 |
| 1973 | 16 | 2 | 0.0625 |
| 1974 | 17 | 5 | 0.15625 |
| 1975 | 18 | 5 | 0.15625 |
| 1976 | 19 | 9 | 0.28125 |
| 1977 | 20 | 7 | 0.21875 |
| 1978 | 21 | 11 | 0.34375 |
| 1979 | 22 | 9 | 0.28125 |
| 1980 | 23 | 6 | 0.1875 |
| 1981 | 24 | 13 | 0.40625 |
| 1982 | 25 | 17 | 0.53125 |
| 1983 | 26 | 18 | 0.5625 |
| 1984 | 27 | 11 | 0.34375 |
| 1985 | 28 | 6 | 0.1875 |
| 1986 | 29 | 4 | 0.125 |
| 1987 | 30 | 8 | 0.25 |
| 1988 | 31 | 9 | 0.28125 |
| 1989 | 32 | 6 | 0.1875 |
| 1990 | 33 | 7 | 0.21875 |
| 1991 | 34 | 4 | 0.125 |
| 1992 | 35 | 3 | 0.09375 |
| 1993 | 36 | 12 | 0.375 |
| 1994 | 37 | 8 | 0.25 |
| 1995 | 38 | 10 | 0.3125 |
| 1996 | 39 | 9 | 0.28125 |
| 1997 | 40 | 9 | 0.28125 |
| 1998 | 41 | 16 | 0.5 |
| 1999 | 42 | 9 | 0.28125 |
| 2000 | 43 | 16 | 0.5 |
| 2001 | 44 | 11 | 0.34375 |
| 2002 | 45 | 12 | 0.375 |
| 2003 | 46 | 15 | 0.46875 |
| 2004 | 47 | 20 | 0.625 |
| 2005 | 48 | 28 | 0.875 |
| 2006 | 49 | 25 | 0.78125 |
| 2007 | 50 | 19 | 0.59375 |
| 2008 | 51 | 11 | 0.34375 |
| 2009 | 52 | 18 | 0.5625 |
| 2010 | 53 | 18 | 0.5625 |
| 2011 | 54 | 16 | 0.5 |
| 2012 | 55 | 22 | 0.6875 |
| 2013 | 56 | 20 | 0.625 |
| 2014 | 57 | 32 | 1 |
| 2015 | 58 | 18 | 0.5625 |
| 2016 | 59 | 24 | 0.75 |

**Minitab 18™ summary for logistic growth fit of text to speech records from *Science Citation Index™***

**Method**

| Algorithm | Marquardt |
| --- | --- |
| Max iterations | 200 |
| Tolerance | 0.00001 |

**Equation**

Text to speech records from SCI = 1 / (1 + 15.5817 * EXP(-0.0595175 * 'Year-Initial_Year'))

**Summary**

| Iterations | 18 |
| --- | --- |
| Final SSE | 0.981584 |
| DFE | 57 |
| MSE | 0.0172208 |
| S | 0.131228 |

**Table A2.9.2**: Retrieved records of text to speech from INSPEC™

| Year | Year-Initial year | Records | Normalized records |
| --- | --- | --- | --- |
| 1969 | 0 | 10 | 0.083333333 |
| 1970 | 1 | 4 | 0.033333333 |
| 1971 | 2 | 10 | 0.083333333 |
| 1972 | 3 | 5 | 0.041666667 |
| 1973 | 4 | 3 | 0.025 |
| 1974 | 5 | 4 | 0.033333333 |
| 1975 | 6 | 6 | 0.05 |
| 1976 | 7 | 11 | 0.091666667 |
| 1977 | 8 | 6 | 0.05 |
| 1978 | 9 | 19 | 0.158333333 |
| 1979 | 10 | 15 | 0.125 |
| 1980 | 11 | 30 | 0.25 |
| 1981 | 12 | 72 | 0.6 |
| 1982 | 13 | 58 | 0.483333333 |
| 1983 | 14 | 59 | 0.491666667 |
| 1984 | 15 | 49 | 0.408333333 |
| 1985 | 16 | 37 | 0.308333333 |
| 1986 | 17 | 64 | 0.533333333 |
| 1987 | 18 | 51 | 0.425 |
| 1988 | 19 | 58 | 0.483333333 |
| 1989 | 20 | 75 | 0.625 |
| 1990 | 21 | 45 | 0.375 |
| 1991 | 22 | 48 | 0.4 |
| 1992 | 23 | 34 | 0.283333333 |
| 1993 | 24 | 45 | 0.375 |
| 1994 | 25 | 74 | 0.616666667 |
| 1995 | 26 | 26 | 0.216666667 |
| 1996 | 27 | 71 | 0.591666667 |
| 1997 | 28 | 38 | 0.316666667 |
| 1998 | 29 | 58 | 0.483333333 |
| 1999 | 30 | 37 | 0.308333333 |
| 2000 | 31 | 63 | 0.525 |
| 2001 | 32 | 36 | 0.3 |
| 2002 | 33 | 73 | 0.608333333 |
| 2003 | 34 | 52 | 0.433333333 |
| 2004 | 35 | 59 | 0.491666667 |
| 2005 | 36 | 56 | 0.466666667 |
| 2006 | 37 | 74 | 0.616666667 |
| 2007 | 38 | 63 | 0.525 |
| 2008 | 39 | 90 | 0.75 |
| 2009 | 40 | 85 | 0.708333333 |
| 2010 | 41 | 118 | 0.983333333 |
| 2011 | 42 | 91 | 0.758333333 |
| 2012 | 43 | 97 | 0.808333333 |
| 2013 | 44 | 108 | 0.9 |
| 2014 | 45 | 120 | 1 |
| 2015 | 46 | 99 | 0.825 |
| 2016 | 47 | 102 | 0.85 |

**Minitab 18™ summary for logistic growth fit of text to speech records from *INSPEC™***

**Method**

| Algorithm | Marquardt |
| --- | --- |
| Max iterations | 200 |
| Tolerance | 0.00001 |

**Equation**

Text to speech records from *INSPEC™* = 1 / (1 + 7.4376 * EXP(-0.0717269 * 'Year-Initial_Year'))

**Summary**

| Iterations | 13 |
| --- | --- |
| Final SSE | 1.04238 |
| DFE | 46 |
| MSE | 0.0226603 |
| S | 0.150534 |

**Table A2.9.3**: Retrieved records of text to speech from Patseer™

| Year | Year-Initial year | Records | Normalized records |
| --- | --- | --- | --- |
| 1961 | 0 | 1 | 0.008695652 |
| 1962 | 1 | 1 | 0.008695652 |
| 1967 | 6 | 4 | 0.034782609 |
| 1968 | 7 | 4 | 0.034782609 |
| 1969 | 8 | 2 | 0.017391304 |
| 1970 | 9 | 3 | 0.026086957 |
| 1971 | 10 | 4 | 0.034782609 |
| 1972 | 11 | 4 | 0.034782609 |
| 1973 | 12 | 1 | 0.008695652 |
| 1974 | 13 | 3 | 0.026086957 |
| 1975 | 14 | 2 | 0.017391304 |
| 1976 | 15 | 1 | 0.008695652 |
| 1977 | 16 | 2 | 0.017391304 |
| 1978 | 17 | 6 | 0.052173913 |
| 1979 | 18 | 4 | 0.034782609 |
| 1980 | 19 | 15 | 0.130434783 |
| 1981 | 20 | 15 | 0.130434783 |
| 1982 | 21 | 12 | 0.104347826 |
| 1983 | 22 | 10 | 0.086956522 |
| 1984 | 23 | 5 | 0.043478261 |
| 1985 | 24 | 10 | 0.086956522 |
| 1986 | 25 | 10 | 0.086956522 |
| 1987 | 26 | 10 | 0.086956522 |
| 1988 | 27 | 25 | 0.217391304 |
| 1989 | 28 | 4 | 0.034782609 |
| 1990 | 29 | 8 | 0.069565217 |
| 1991 | 30 | 12 | 0.104347826 |
| 1992 | 31 | 29 | 0.252173913 |
| 1993 | 32 | 27 | 0.234782609 |
| 1994 | 33 | 32 | 0.27826087 |
| 1995 | 34 | 31 | 0.269565217 |
| 1996 | 35 | 38 | 0.330434783 |
| 1997 | 36 | 46 | 0.4 |
| 1998 | 37 | 62 | 0.539130435 |
| 1999 | 38 | 59 | 0.513043478 |
| 2000 | 39 | 64 | 0.556521739 |
| 2001 | 40 | 76 | 0.660869565 |
| 2002 | 41 | 90 | 0.782608696 |
| 2003 | 42 | 105 | 0.913043478 |
| 2004 | 43 | 115 | 1 |
| 2005 | 44 | 102 | 0.886956522 |
| 2006 | 45 | 88 | 0.765217391 |
| 2007 | 46 | 84 | 0.730434783 |
| 2008 | 47 | 90 | 0.782608696 |
| 2009 | 48 | 64 | 0.556521739 |
| 2010 | 49 | 48 | 0.417391304 |
| 2011 | 50 | 61 | 0.530434783 |
| 2012 | 51 | 78 | 0.67826087 |
| 2013 | 52 | 62 | 0.539130435 |
| 2014 | 53 | 62 | 0.539130435 |
| 2015 | 54 | 89 | 0.773913043 |
| 2016 | 55 | 37 | 0.32173913 |

**Minitab 18™ summary for logistic growth fit of text to speech records from *Patseer™***

**Method**

| Algorithm | Marquardt |
| --- | --- |
| Max iterations | 200 |
| Tolerance | 0.00001 |

**Equation**

Text to speech records from *Patseer™* = 1 / (1 + 71.7059 * EXP(-0.106548 * 'Year-Initial_Year'))

**Summary**

| Iterations | 28 |
| --- | --- |
| Final SSE | 1.23996 |
| DFE | 50 |
| MSE | 0.0247992 |
| S | 0.157478 |

**Table A2.9.4**: Retrieved records of text to speech from Factiva™

| Year | Year-Initial year | Records | Normalized records |
| --- | --- | --- | --- |
| 1981 | 0 | 1 | 0.016129 |
| 1982 | 1 | 1 | 0.016129 |
| 1983 | 2 | 1 | 0.016129 |
| 1984 | 3 | 3 | 0.048387 |
| 1985 | 4 | 1 | 0.016129 |
| 1988 | 7 | 2 | 0.032258 |
| 1989 | 8 | 2 | 0.032258 |
| 1990 | 9 | 4 | 0.064516 |
| 1991 | 10 | 1 | 0.016129 |
| 1992 | 11 | 9 | 0.145161 |
| 1993 | 12 | 20 | 0.322581 |
| 1994 | 13 | 16 | 0.258065 |
| 1995 | 14 | 26 | 0.419355 |
| 1996 | 15 | 43 | 0.693548 |
| 1997 | 16 | 28 | 0.451613 |
| 1998 | 17 | 34 | 0.548387 |
| 1999 | 18 | 34 | 0.548387 |
| 2000 | 19 | 62 | 1 |
| 2001 | 20 | 53 | 0.854839 |
| 2002 | 21 | 60 | 0.967742 |
| 2003 | 22 | 53 | 0.854839 |
| 2004 | 23 | 33 | 0.532258 |
| 2005 | 24 | 12 | 0.193548 |
| 2006 | 25 | 16 | 0.258065 |
| 2007 | 26 | 12 | 0.193548 |
| 2008 | 27 | 3 | 0.048387 |
| 2009 | 28 | 12 | 0.193548 |
| 2010 | 29 | 21 | 0.33871 |
| 2011 | 30 | 33 | 0.532258 |
| 2012 | 31 | 16 | 0.258065 |
| 2013 | 32 | 11 | 0.177419 |
| 2014 | 33 | 33 | 0.532258 |
| 2015 | 34 | 36 | 0.580645 |
| 2016 | 35 | 55 | 0.887097 |

**Minitab 18™ summary for hype-type evolution fit of text to speech records from *Factiva™***

**Method**

| Algorithm | Marquardt |
| --- | --- |
| Max iterations | 200 |
| Tolerance | 0.0001 |

**Equation**

Text to speech from *Factiva™* = 0.0241538 * 1.02133 * 1 * EXP(1.02133 * 'Year-Initial_Year') / ((0.0241538 + EXP(1.02133 * 'Year-Initial_Year')) ^ 2) + 0.504395 * 1 / (1 + 0.0241538 * EXP(-1.02133 * (Year - 15.6144 - 1981)))

**Summary**

| Iterations | 34 |
| --- | --- |
| Final SSE | 1.69184 |
| DFE | 30 |
| MSE | 0.0563946 |
| S | 0.237475 |

**A2.10 Wireless Local Area Network**

**Table A2.10.1**: Retrieved records of wireless local area network from Science Citation Index™

| Year | Year-Initial year | Records | Normalized records |
| --- | --- | --- | --- |
| 1989 | 0 | 1 | 0.003412969 |
| 1993 | 4 | 10 | 0.034129693 |
| 1994 | 5 | 3 | 0.010238908 |
| 1995 | 6 | 3 | 0.010238908 |
| 1996 | 7 | 16 | 0.054607509 |
| 1997 | 8 | 21 | 0.071672355 |
| 1998 | 9 | 18 | 0.061433447 |
| 1999 | 10 | 14 | 0.04778157 |
| 2000 | 11 | 46 | 0.156996587 |
| 2001 | 12 | 35 | 0.119453925 |
| 2002 | 13 | 63 | 0.215017065 |
| 2003 | 14 | 140 | 0.4778157 |
| 2004 | 15 | 174 | 0.593856655 |
| 2005 | 16 | 252 | 0.860068259 |
| 2006 | 17 | 221 | 0.754266212 |
| 2007 | 18 | 253 | 0.863481229 |
| 2008 | 19 | 232 | 0.791808874 |
| 2009 | 20 | 243 | 0.829351536 |
| 2010 | 21 | 220 | 0.750853242 |
| 2011 | 22 | 248 | 0.846416382 |
| 2012 | 23 | 221 | 0.754266212 |
| 2013 | 24 | 242 | 0.825938567 |
| 2014 | 25 | 266 | 0.907849829 |
| 2015 | 26 | 293 | 1 |
| 2016 | 27 | 259 | 0.883959044 |

**Minitab 18™ summary for logistic growth fit of Wireless Local Area Network records from *Science Citation Index™***

**Method**

| Algorithm | Marquardt |
| --- | --- |
| Max iterations | 200 |
| Tolerance | 0.00001 |

**Equation**

WLAN records from SCI = 1 / (1 + 441.147 * EXP(-0.413397 * 'Year-Initial_Year'))

**Summary**

| Iterations | 31 |
| --- | --- |
| Final SSE | 0.242890 |
| DFE | 23 |
| MSE | 0.0105605 |
| S | 0.102764 |

**Table A2.10.2**: Retrieved records of wireless local area network from INSPEC™

| Year | Year-Initial year | Records | Normalized records |
| --- | --- | --- | --- |
| 1982 | 0 | 1 | 0.001041667 |
| 1987 | 5 | 1 | 0.001041667 |
| 1990 | 8 | 1 | 0.001041667 |
| 1991 | 9 | 1 | 0.001041667 |
| 1992 | 10 | 10 | 0.010416667 |
| 1993 | 11 | 23 | 0.023958333 |
| 1994 | 12 | 23 | 0.023958333 |
| 1995 | 13 | 29 | 0.030208333 |
| 1996 | 14 | 46 | 0.047916667 |
| 1997 | 15 | 58 | 0.060416667 |
| 1998 | 16 | 59 | 0.061458333 |
| 1999 | 17 | 95 | 0.098958333 |
| 2000 | 18 | 117 | 0.121875 |
| 2001 | 19 | 139 | 0.144791667 |
| 2002 | 20 | 318 | 0.33125 |
| 2003 | 21 | 549 | 0.571875 |
| 2004 | 22 | 723 | 0.753125 |
| 2005 | 23 | 853 | 0.888541667 |
| 2006 | 24 | 937 | 0.976041667 |
| 2007 | 25 | 960 | 1 |
| 2008 | 26 | 937 | 0.976041667 |
| 2009 | 27 | 906 | 0.94375 |
| 2010 | 28 | 876 | 0.9125 |
| 2011 | 29 | 878 | 0.914583333 |
| 2012 | 30 | 816 | 0.85 |
| 2013 | 31 | 855 | 0.890625 |
| 2014 | 32 | 918 | 0.95625 |
| 2015 | 33 | 873 | 0.909375 |
| 2016 | 34 | 869 | 0.905208333 |

**Minitab 18™ summary for logistic growth fit of Wireless Local Area Network records from *INSPEC™***

**Method**

| Algorithm | Marquardt |
| --- | --- |
| Max iterations | 200 |
| Tolerance | 0.00001 |

**Equation**

WLAN records from *INSPEC™* = 1 / (1 + 4.82104e+007 * EXP(-0.85439 * 'Year-Initial_Year'))

**Summary**

| Iterations | 65 |
| --- | --- |
| Final SSE | 0.0882834 |
| DFE | 27 |
| MSE | 0.0032698 |
| S | 0.0571818 |

**Table A2.10.3**: Retrieved records of wireless local area network from Patseer™

| Year | Year-Initial year | Records | Normalized records |
| --- | --- | --- | --- |
| 1991 | 0 | 1 | 0.000690131 |
| 1992 | 1 | 2 | 0.001380262 |
| 1993 | 2 | 4 | 0.002760524 |
| 1994 | 3 | 16 | 0.011042098 |
| 1995 | 4 | 16 | 0.011042098 |
| 1996 | 5 | 18 | 0.01242236 |
| 1997 | 6 | 12 | 0.008281573 |
| 1998 | 7 | 17 | 0.011732229 |
| 1999 | 8 | 29 | 0.020013803 |
| 2000 | 9 | 66 | 0.045548654 |
| 2001 | 10 | 163 | 0.112491373 |
| 2002 | 11 | 442 | 0.305037957 |
| 2003 | 12 | 593 | 0.409247757 |
| 2004 | 13 | 623 | 0.429951691 |
| 2005 | 14 | 743 | 0.512767426 |
| 2006 | 15 | 637 | 0.439613527 |
| 2007 | 16 | 449 | 0.309868875 |
| 2008 | 17 | 385 | 0.265700483 |
| 2009 | 18 | 383 | 0.264320221 |
| 2010 | 19 | 441 | 0.304347826 |
| 2011 | 20 | 534 | 0.368530021 |
| 2012 | 21 | 713 | 0.492063492 |
| 2013 | 22 | 1048 | 0.723257419 |
| 2014 | 23 | 1145 | 0.790200138 |
| 2015 | 24 | 1449 | 1 |
| 2016 | 25 | 1236 | 0.85300207 |

**Minitab 18™ summary for logistic growth fit of Wireless Local Area Network records from *Patseer™***

**Method**

| Algorithm | Marquardt |
| --- | --- |
| Max iterations | 200 |
| Tolerance | 0.00001 |

**Equation**

WLAN records from *Patseer™* = 1 / (1 + 44.2856 * EXP(-0.201577 * 'Year-Initial_Year'))

**Summary**

| Iterations | 15 |
| --- | --- |
| Final SSE | 0.438535 |
| DFE | 24 |
| MSE | 0.0182723 |
| S | 0.135175 |

**Table A2.10.4**: Retrieved records of wireless local area network from Factiva™

| Year | Year-Initial year | Records | Normalized records |
| --- | --- | --- | --- |
| 1988 | 0 | 1 | 0.000683 |
| 1989 | 1 | 7 | 0.004778 |
| 1990 | 2 | 14 | 0.009556 |
| 1991 | 3 | 23 | 0.0157 |
| 1992 | 4 | 43 | 0.029352 |
| 1993 | 5 | 62 | 0.042321 |
| 1994 | 6 | 81 | 0.05529 |
| 1995 | 7 | 105 | 0.071672 |
| 1996 | 8 | 136 | 0.092833 |
| 1997 | 9 | 145 | 0.098976 |
| 1998 | 10 | 191 | 0.130375 |
| 1999 | 11 | 181 | 0.123549 |
| 2000 | 12 | 198 | 0.135154 |
| 2001 | 13 | 425 | 0.290102 |
| 2002 | 14 | 837 | 0.571331 |
| 2003 | 15 | 1458 | 0.995222 |
| 2004 | 16 | 1047 | 0.714676 |
| 2005 | 17 | 499 | 0.340614 |
| 2006 | 18 | 328 | 0.223891 |
| 2007 | 19 | 235 | 0.16041 |
| 2008 | 20 | 305 | 0.208191 |
| 2009 | 21 | 413 | 0.281911 |
| 2010 | 22 | 511 | 0.348805 |
| 2011 | 23 | 589 | 0.402048 |
| 2012 | 24 | 860 | 0.587031 |
| 2013 | 25 | 876 | 0.597952 |
| 2014 | 26 | 1375 | 0.938567 |
| 2015 | 27 | 1465 | 1 |
| 2016 | 28 | 1344 | 0.917406 |

**Minitab 18™ summary for hype-type evolution fit of Wireless Local Area Network records from *Factiva™***

**Method**

| Algorithm | Marquardt |
| --- | --- |
| Max iterations | 200 |
| Tolerance | 0.0001 |

**Equation**

WLAN from *Factiva™* = 18385.5 * 1.14478 * 1 * EXP(1.14478 * 'Year-Initial_Year') / ((18385.5 + EXP(1.14478 * 'Year-Initial_Year')) ^ 2) + 0.553448 * 1 / (1 + 18385.5 * EXP(-1.14478 * (Year - 3.98422 - 1988)))

**Summary**

| Iterations | 89 |
| --- | --- |
| Final SSE | 1.39083 |
| DFE | 25 |
| MSE | 0.0556333 |
| S | 0.235867 |

**Appendix 3.** Outliers test for estimating a less biased acceptance threshold for S (ATS)

**Method**

| Null hypothesis | All data values come from the same normal population |
| --- | --- |
| Alternative hypothesis | Smallest or largest data value is an outlier |
| Significance level | α = 0.05 |

**Grubbs' Test**

| Variable | N | Mean | StDev | Min | Max | G | P |
| --- | --- | --- | --- | --- | --- | --- | --- |
| *Science Citation Index™* | 10 | 0.0829 | 0.0529 | 0.0352 | 0.2050 | 2.31 | 0.045 |
| *INSPEC™* | 10 | 0.0853 | 0.0412 | 0.0141 | 0.1505 | 1.73 | 0.627 |
| *Patseer™* | 10 | 0.1304 | 0.0348 | 0.0773 | 0.1798 | 1.53 | 1.000 |
| *Factiva™* | 10 | 0.1399 | 0.0889 | 0.0300 | 0.2570 | 1.32 | 1.000 |

**Outlier**

| Variable | Row | Outlier |
| --- | --- | --- |
| *Science Citation Index™* | 3 | 0.205 |

**Appendix 4**. Prediction intervals for each *Science Citation Index™*, *INSPEC™*, *Patseer™* and *Factiva™*

**Prediction Interval for a Single Future Observation in *Science Citation Index™***

Future Sample Size: 1

Estimated Value: 0.0693172

95% Prediction Interval: (-0.0106, 0.1492)

**Prediction Interval for a Single Future Observation in *INSPEC™***

Future Sample Size: 1

Estimated Value: 0.0852558

95% Prediction Interval: (-0.0125, 0.1830)

**Prediction Interval for a Single Future Observation in *Patseer™***

Future Sample Size: 1

Estimated Value: 0.130387

95% Prediction Interval: (0.0478, 0.2130)

**Prediction Interval for a Single Future Observation in *Factiva™***

Future Sample Size: 1

Estimated Value: 0.139899

95% Prediction Interval: (-0.0711, 0.3509)

**Appendix 5.** Search queries for AM technologies

**Table A5.1** Search queries for AM technologies on *Science Citation Index™* and *INSPEC™* databases

| Technology | Search query |
| --- | --- |
| Binder jetting | TI= ("Binder-jet*" OR Voxeljet) AND (TS=((Additive) NEAR/1 (Manufactur*)) OR TS=((3D OR 3-Dimensional OR three-D OR three-dimensional) NEAR/1 (Print*))) |
| Directed energy deposition | TI=("Direct* energy deposition" OR "Laser clad*" OR "Laser-engineer* net shaping" OR ((Laser OR Direct) NEAR/1 (Metal-deposition)) OR "Laser freeform-fabrication" OR "Laser direct-casting" OR Laser-consolidation OR ((Direct*) NEAR/1 Light fabrication)) AND (TS=((Additive) NEAR/1 (Manufactur*)) OR TS=((3D OR 3-Dimensional OR three-D OR three-dimensional) NEAR/1 (Print*))) |
| Material extrusion | TI=("Material extrusion" OR "Fuse* filament-fabricat*" OR "Fuse* deposition-model*" OR "Fuse* layer* model*" OR "Plastic jet-print*") AND (TS=((Additive) NEAR/1 (Manufactur*)) OR TS=((3D OR 3-Dimensional OR three-D OR three-dimensional) NEAR/1 (Print*))) |
| Material jetting | TI=(("Material jet*" OR (Multijet OR Multi-jet) NEAR/1 model*) OR Thermojet OR (Inkjet OR Ink-jet) NEAR/1 print*) AND (TS=((Additive) NEAR/1 (Manufactur*)) OR TS=((3D OR 3-Dimensional OR three-D OR three-dimensional) NEAR/1 (Print*))) |
| Powder bed fusion | TI=("Powder bed fusion" OR "Direct-metal laser sinter*" OR ("Selective laser" OR "Electron beam") NEAR/1 (Melt* OR Sinter*)) AND (TS=((Additive) NEAR/1 (Manufactur*)) OR TS=((3D OR 3-Dimensional OR three-D OR three-dimensional) NEAR/1 (Print*))) |
| Sheet lamination | TI=("Sheet laminat*" OR (Ultrasonic NEAR/1 (Consolidat* OR "Additive manufactur*")) OR "Lamination object manufactur*") AND (TS=((Additive) NEAR/1 (Manufactur*)) OR TS=((3D OR 3-Dimensional OR three-D OR three-dimensional) NEAR/1 (Print*))) |
| Vat photopolymerization | TI=("Vat photopolymerizat*" OR Stereolithograph* OR SLA OR "Thin-film photopolymerizat*") AND (TS=((Additive) NEAR/1 (Manufactur*)) OR TS=((3D OR 3-Dimensional OR three-D OR three-dimensional) NEAR/1 (Print*))) |

**Table A5.2** Search queries for AM technologies on *Patseer™*

| Technology | Search query |
| --- | --- |
| Binder jetting | T: (Binder-jet* OR Voxeljet) AND A: ((3D OR 3-Dimensional OR three-D OR three-dimensional) WD1 (Print*) OR Additive manufactur*) AND PTYP:("Patent" OR "Application") |
| Directed energy deposition | T: (Direct* energy deposition OR Laser clad* OR Laser-engineer* net shaping OR ((Laser OR Direct) WD/1 (Metal-deposition)) OR Laser freeform-fabrication OR Laser direct-casting OR Laser-consolidation OR ((Direct*) wd/1 Light fabrication)) AND A: ((3D OR 3-Dimensional OR three-D OR three-dimensional) WD1 (Print*) OR Additive manufactur*) AND PTYP:("Patent" OR "Application") |
| Material extrusion | T: (Material extrusion OR Fuse* filament fabricati* OR Fuse* deposition model* OR Fuse* layer* model* OR Plastic jet print*) AND A: ((3D OR 3-Dimensional OR three-D OR three-dimensional) WD1 (Print*) OR Additive manufactur*) AND PTYP:("Patent" OR "Application") |
| Material jetting | T: ((Material-jet* OR (Multijet OR Multi-jet) WD1 model*) OR Thermojet OR (Inkjet OR Ink-jet) WD1 print*) AND A: ((3D OR 3-Dimensional OR three-D OR three-dimensional) WD1 (Print*) OR Additive manufactur*) AND PTYP:("Patent" OR "Application") |
| Powder bed fusion | T:(Powder bed fusion OR Direct metal laser sinter* OR (Selective laser OR Electron beam) wd1 (Melt* OR Sinter*)) AND A: ((3D OR 3-Dimensional OR three-D OR three-dimensional) WD1 (Print*) OR Additive manufactur*) AND PTYP:("Patent" OR "Application") |
| Sheet lamination | T: (Sheet-laminat* OR (Ultrasonic WD1 (Consolidat* OR Additive-manufactur*)) OR Lamination object manufactur*) AND A: ((3D OR 3-Dimensional OR three-D OR three-dimensional) WD1 (Print*) OR Additive manufactur*) AND PTYP:("Patent" OR "Application") |
| Vat photopolymerization | T: (Vat photopolymerizat* OR Stereolithograph* OR SLA OR Thin-film photopolymerizat*) AND A: ((3D OR 3-Dimensional OR three-D OR three-dimensional) WD1 (Print*) OR Additive manufactur*) AND PTYP:("Patent" OR "Application") |
|  | |

**Table A5.3** Search queries for AM technologies on *Factiva™*

| Technology | Search query |
| --- | --- |
| Binder jetting | (3D print* OR three-D print* OR 3 dimensional print* OR three dimensional print* OR additive manufactur*) AND (Binder-jet* OR Voxeljet) |
| Directed energy deposition | (3D print* OR three-D print* OR 3 dimensional print* OR three dimensional print* OR additive manufactur*) AND (Direct* energy deposition OR Laser clad* OR Laser-engineer* net shaping OR ((Laser OR Direct) WD/1 (Metal-deposition)) OR Laser freeform-fabrication OR Laser direct-casting OR Laser-consolidation OR ((Direct*) wd/1 Light fabrication)) |
| Material extrusion | (3D print* OR three-D print* OR 3 dimensional print* OR three dimensional print* OR additive manufactur*) AND (Material extrusion OR Fuse* filament fabricat* OR Fuse* deposition model* OR Fuse* layer* model* OR Plastic jet print*) |
| Material jetting | (3D print* OR three-D print* OR 3 dimensional print* OR three dimensional print* OR additive manufactur*) AND ((Material-jet* OR (Multijet OR Multi-jet) NEAR1 model*) OR Thermojet OR (Inkjet OR Ink-jet) NEAR1 print*) |
| Powder bed fusion | (3D print* OR three-D print* OR 3 dimensional print* OR three dimensional print* OR additive manufactur*) AND (Powder bed fusion OR Direct metal laser sinter* OR Selective laser melt* OR selective laser sinter* OR electron beam melt* OR electron beam sinter*) |
| Sheet lamination | (3D print* OR three-D print* OR 3 dimensional print* OR three dimensional print* OR additive manufactur*) AND (Sheet-laminat* OR (Ultrasonic NEAR1 (Consolidat* OR Additive-manufactur*)) OR Lamination object manufactur*) |
| Vat photopolymerization | (3D print* OR three-D print* OR 3 dimensional print* OR three dimensional print* OR additive manufactur*) AND (Vat photopolymerizat* OR Stereolithograph* OR SLA OR Thin-film photopolymerizat*) |
|  | |
